# Artificial intelligence for prediction of atrial fibrillation in the stroke unit: a retrospective derivation validation cohort study

**DOI:** 10.1016/j.ebiom.2025.105869

**Published:** 2025-08-05

**Authors:** Maximilian Schoels, Laura Krumm, Alexander Nelde, Manuel C. Olma, Christian H. Nolte, Jan F. Scheitz, Markus G. Klammer, Christoph Leithner, Andreas Meisel, Franziska Scheibe, Michael Krämer, Karl Georg Haeusler, Matthias Endres, Christian Meisel

**Affiliations:** a Computational Neurology, Department of Neurology, Charité – Universitätsmedizin Berlin, Germany; b Berlin Institute of Health, Berlin, Germany; c Center for Stroke Research Berlin, Berlin, Germany; d Bernstein Center for Computational Neuroscience, Berlin, Germany; e Einstein Center for Neurosciences, Berlin, Germany; f Institute for Theoretical Biology, Department of Biology, Humboldt-Universität zu Berlin, Berlin, Germany; g Department of Neurology with Experimental Neurology, Charité – Universitätsmedizin Berlin, Germany; h German Center for Neurodegenerative Diseases (DZNE), Partner Site Berlin, Germany; i German Center for Cardiovascular Research (DZHK), Partner Site Berlin, Germany; j NeuroCure Cluster of Excellence, Charité – Universitätsmedizin Berlin, Berlin, Germany; k Neuroscience Clinical Research Center, Berlin, Germany; l Department of Neurology, Universitätsklinikum Ulm, Ulm, Germany

**Keywords:** Stroke, Atrial fibrillation, Prediction, Machine learning, Artificial intelligence, Heart rate variability

## Abstract

**Background:**

Paroxysmal atrial fibrillation (AF) is a major cause of stroke but is often undetected in routine clinical practice. Effective stratification is needed to identify patients with stroke who might benefit the most from intensified AF screening. Several artificial intelligence models have been proposed to predict AF based on ECG in sinus rhythm, but broad implementation has been limited. The most valuable input features and most effective model design for AF prediction are also unclear.

**Methods:**

We developed and tested AF prediction models utilising continuous electrocardiogram monitoring (CEM) recordings from the first 72 h after admission and multiple clinical input features from patients with stroke hospitalised at Charité, Berlin, Germany, between September 2020 and August 2023. We compared different models and input data to identify the best-performing model for prediction of AF. The relative contributions of different input data sources were assessed for explainability. A final model was externally validated using the first hour of monitoring data from the intervention group of the prospective multicentre MonDAFIS study.

**Findings:**

The derivation dataset included 2068 patients with acute ischaemic stroke, of whom 469 (22.7%) had AF, first detected before or during the index hospital stay (366 vs. 103). In predicting newly detected AF, a Bayesian fusion model emerged as best, achieving a ROC-AUC of 0.89 (95% CI: 0.80, 0.96). Model introspection indicated that HRV was the main driver of the model's predictions. A final, simplified tree-based ensemble model using age and HRV parameters of the first hour of CEM data achieved similar performance (ROC-AUC 0.88, 95% CI: 0.79, 0.95). The final model consistently outperformed the AS5F score in a real-world scenario external validation on the MonDAFIS dataset (1519 patients, thereof 36 (2.37%) with AF; ROC-AUC 0.79 vs. ROC-AUC 0.69, p = 4.69e-03).

**Interpretation:**

HRV appears to be the most informative variable for predicting AF. A computationally inexpensive model requiring only 1 h of single-lead CEM data and patients' age supports prediction of AF after acute ischaemic stroke for up to seven days. Such a model may enable risk-based stratification for cardiac monitoring, prioritising efforts where most needed to enhance AF screening efficiency and, ultimately, secondary stroke prevention.

**Funding:**

This study was supported by the 10.13039/501100002347German Federal Ministry of Education and Research and the 10.13039/501100001659German Research Foundation.

**Research in context**

**Evidence before this study**

We searched PubMed without language restrictions for articles published from database inception to March 28, 2025, using the keywords: (“stroke”) AND (“atrial fibrillation”) AND (“risk stratification” OR “prediction”) AND (“neural network” OR “machine learning” OR “artificial intelligence”). This search yielded 182 articles. A second query, in which (“neural network” OR “machine learning” OR “artificial intelligence”) was replaced with (“sinus”), returned 144 results. After removing duplicates, 307 unique articles remained. By screening titles and/or abstracts, we identified 11 publications that reported on the development and validation of new models for AF prediction for patients with ischaemic stroke. An additional seven publications were identified through screening of reviews, reference lists of the above publications, and free-text searches.

Of the 18 identified studies, nine developed multimodal AF prediction algorithms, three of which incorporated ECG features to varying extents. Six studies focused on the analysis of 12-lead ECGs in sinus rhythm; three of these also incorporated additional clinical data. Two of the six studies validated the same previously reported AI-based model. Notably, all six models were initially trained on ECG data from non-stroke populations. Three studies validated the same commercially available heart rate variability-based prediction algorithm, using continuous electrocardiogram monitoring (CEM) data. Most studies assessed the relative contribution of different data sources to varying degrees; however, in-depth analyses—such as SHapley Additive exPlanations (SHAP) interpretation or formal comparative testing—were rarely reported. Direct comparisons between analysis of 12-lead ECG analysis and CEM were not conducted.

A variety of conventional risk scores have been validated for AF prediction after stroke, including AS5F, CHASE-LESS, (Re-)CHARGE-AF, C2HEST, HATCH, HAVOC, and CHA_2_DS_2_-VASc. The advantages and limitations of these scores have been discussed extensively elsewhere. By design, these models do not incorporate ECG features and are inherently static, limiting their ability to capture dynamic or electrophysiological indicators of AF risk. Expanding the search to include studies on patients without stroke reveals several models for atrial fibrillation prediction based on ECG. However, due to significant differences in disease burden and baseline characteristics, these models are not readily transferable to patients with ischaemic stroke. Moreover, most rely on 12-lead ECG analysis, which may limit their feasibility for automated background analysis in the stroke unit context.

Thus, the optimal model architecture and input feature combination for AFDAS prediction remains undefined. Moreover, many existing models are proof-of-concept and not yet tailored for clinical implementation. These gaps underscore the need for further research to identify effective and clinically applicable approaches for accurate AFDAS prediction.

**Added value of this study**

We developed and evaluated multiple short-term AF prediction models for patients with acute ischaemic stroke or transient ischaemic attack using data from the Charité hospital, Berlin, Germany. These models incorporated various architectures and input features. SHAP analysis and comparative assessments identified HRV as the most informative feature. A simple tree-based ensemble model, utilising only age and beat-to-beat intervals, demonstrated performance almost on par with more complex fusion models that used all available data. This simpler model is easier to implement and well-suited for clinical practice. Upon external validation, the simplified model consistently outperformed the validated AS5F score on datasets from our institution and a larger, independent dataset from the randomised, controlled multi-centre trial MonDAFIS.

**Implications of all the available evidence**

Automated, short-term AF prediction is feasible with good accuracy using only age and beat-to-beat intervals as inputs. This approach allows for an easily implementable screening tool to inform decisions about prolonged cardiac monitoring and may help bridge the gap between the increasing number of prediction models reported in the literature and the lack of robust prediction tools in clinical use.

## Introduction

Acute ischaemic stroke is a common and potentially devastating condition, affecting approximately 7.6 million people worldwide each year.^1^ Despite therapeutic advancements, risk of stroke recurrence is high, varying from 10 to 50% in the subsequent 5 years.^2^ Atrial fibrillation (AF) is a major cause of ischaemic stroke, accounting for about a quarter of all cases.^3^ However, AF may be paroxysmal and clinically asymptomatic. Consequently, it is frequently missed by routine work-up.^4^ Trials evaluating empiric anticoagulation of patients with embolic stroke of undetermined source (ESUS) repeatedly failed.^5^, ^6^, ^7^ Thus, accurate identification of AF remains crucial for secondary stroke prevention.^8^ While the clinical relevance of device-detected AF months after the index event is increasingly being critically discussed in the light of recent studies on patients without stroke,^9^^,^^10^ there is a consensus that patients with ischaemic stroke should undergo continuous electrocardiogram monitoring (CEM) for AF detection in the acute phase of stroke.^4^ However, the ideal CEM duration is unclear. Under the pressure of rising stroke incidences and economic constraints, the decision whether and how long to monitor patients beyond 24 h post-stroke needs to be made in thousands of stroke units every day. Yet, established markers besides the clinician's intuition to guide this decision are missing. Consequently, more effective stratification methods are needed to select those patients with stroke who will most likely benefit from intensified AF screening.^11^

Artificial Intelligence (AI) can predict AF based on sinus rhythm ECG and may offer transformative approaches to stroke work-up.^12^^,^^13^ However, AF prediction studies were done in the general population, and it is not clear whether these methods also work in patients with stroke. Markers for AF in patients with stroke may differ from those in other patient groups for several reasons. First, this population is typically older and suffers from more comorbidities which may alter the features distinguishing patients with and without AF.^14^ Second, the impact of acute ischaemic stroke on the autonomous nervous system might affect cardiac function (heart-brain-axis) and ECG patterns.^15^ Third, the concept of “Atrial Fibrillation Detected after Stroke or TIA” (AFDAS) as a distinct entity of AF merits special consideration since risk factors and ECG-findings differ from those of “classical” AF.^16^ Therefore, AF risk stratification models need to be developed and validated in post-stroke cohorts.

To develop prediction models for the stroke unit, it is crucial to identify which data and which model architectures provide the best AF prediction suitable for broad application. More generally, while deep neural networks (DNN) analysing raw-ECG have proven powerful to predict AF, it remains unclear how much additional information these methods can provide in comparison to more simple and traditional ECG features, including heart rate variability (HRV),^17^^,^^18^ clinical characteristics^19^^,^^20^ or scores.^21^^,^^22^ Within the context of ischaemic stroke, no systematic comparison has yet examined different combinations of input features and model architectures—including DNNs. Thus, the optimal AF-prediction strategy post-stroke remains unclear.

We developed and tested a data warehouse-driven machine learning (ML) model to predict AF in patients with acute ischaemic stroke or transient ischaemic attack. Routine monitoring data from the first 72 h after admission to the stroke unit was used for model derivation. We systematically compared traditional ML models, DNNs and combinations of both using a broad range of multimodal data, including raw CEM data, HRV, laboratory values, patient characteristics, and concomitant diagnoses to identify the best performing model architecture and most informative data features. Thorough analysis was done to ensure explainability of our model's predictions. We validated our best performing model on two out-of-sample datasets.

## Methods

### Rational of the study design

**Fig. 1:**
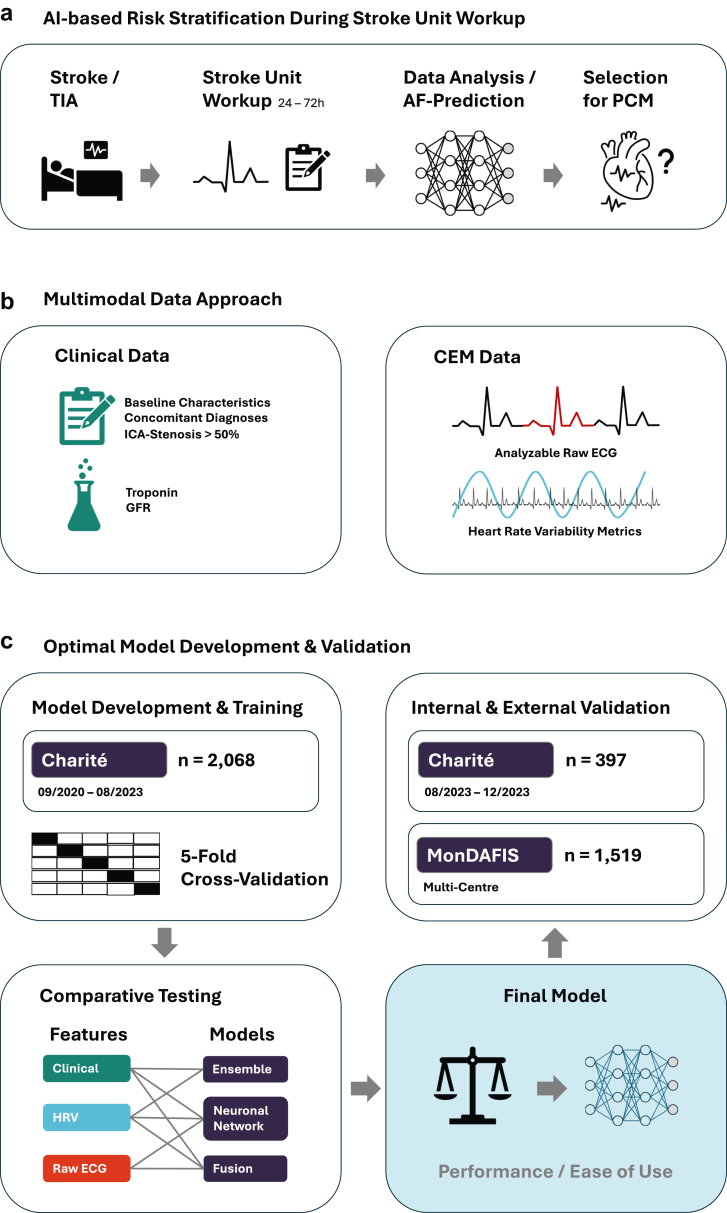
**Study design.** a) AI-based risk stratification during stroke unit work-up. Data routinely collected during stroke unit workup serve as input variables; a ML model automatically stratifies patients according to their individual risk of underlying AF for PCM. b) Multimodal data used during comparative analyses. c) Optimal model development and external validation. Different ML models using different types and combinations of input data and different types of model architecture were systematically explored using 5-fold nested cross-validation. A final model was selected for internal and external validation based on a trade-off between predictive performance and ease-of-use. Sensitivity, positive predictive value, and negative predictive value at a predefined specificity threshold of 90% were calculated to evaluate clinical applicability. AI: Artificial intelligence; TIA: Transient ischaemic attack; AF: Atrial fibrillation; PCM: Prolonged cardiac monitoring; ICA: Internal carotid artery; GFR: Glomerular filtration rate; HRV: Heart rate variability; CEM: Continuous electrocardiogram monitoring; ML: Machine learning.

This study follows a derivation-validation design. Specifically, we developed, systematically compared and tested AF prediction models utilising different models and input data to identify the best-performing approach (Fig. 1). A final model was validated using the first hour of monitoring data from an out-of-sample in-house dataset and the intervention group of the prospective multicentre study. High-quality, fully expert-reviewed long-term Holter-ECG data from patients with ischaemic stroke but without pre-existing AF are rare. Consequently, we opted to use automatically labelled, real-world data for model derivation, reserving the manually curated dataset for final external validation. This approach helps ensure the reliability of the definitive validation results even when working with smaller patient cohorts (as those ultimately diagnosed with AF after stroke by long-term Holter-ECG).

“Standards for Reporting Diagnostic Accuracy Studies” (STARD), “Transparent Reporting of a Multivariable Prediction Model for Individual Prognosis or Diagnosis” (TRIPOD), and “MINimum Information for Medical AI Reporting” (MINIMAR) guidelines were followed in the design of this study and the writing of the manuscript.

### Ethics

The use and analysis of data for model derivation and internal validation were approved by the Institutional Review Board of Charité – Universitätsmedizin Berlin (reference number: EA1/376/20), which waived the requirement for informed consent. External validation data were obtained from the previously published, multicentre MonDAFIS trial (NCT02204267), in which all patients provided written and informed consent.

### Datasets

#### Derivation dataset

We retrospectively analysed all patients diagnosed with acute ischaemic stroke (AIS, ICD-10: I63.∗) or transient ischaemic attack (TIA, ICD-10: G45.∗) treated between September 30, 2020 and August 19, 2023 in one of three stroke units at Charité – Universitätsmedizin Berlin, Germany (consecutive series). 24 monitoring beds allowed data transfer and integration into the Data Warehouse Connect system (DWC, Philips) for long-term storage of monitoring data. The Charité/Berlin Institute of Health (BIH) Health Data Lake (HDL), a Hadoop based platform that allows storage of a multitude of clinical, epidemiological, laboratory, and monitoring data, was used for further data integration, harmonisation, and analysis.

CEM data, sampled at 500 Hz during the first 72 h following hospital admission, were collected for all patients. For the purposes of morphologic CEM analysis, we focused on lead II, as it is commonly available in clinical settings and facilitates broad applicability.^23^ In addition to raw CEM signals, processed CEM data containing individual heartbeats labelled by a proprietary Philips algorithm and RR intervals were stored and utilised for HRV analysis. The Philips monitoring system includes an internal AF detection algorithm, which evaluates RR interval irregularity, PR interval variability, and P-wave morphology. The system also records alarm metadata, including the type of alert (e.g., AF alert), as well as onset and announcement times; all were captured and stored in the DWC. Beyond CEM metrics, the HDL also integrated a comprehensive array of patient-specific parameters, including blood pressure measurements, laboratory test results, clinical scores, and comorbid diagnoses.

**Fig. 2:**
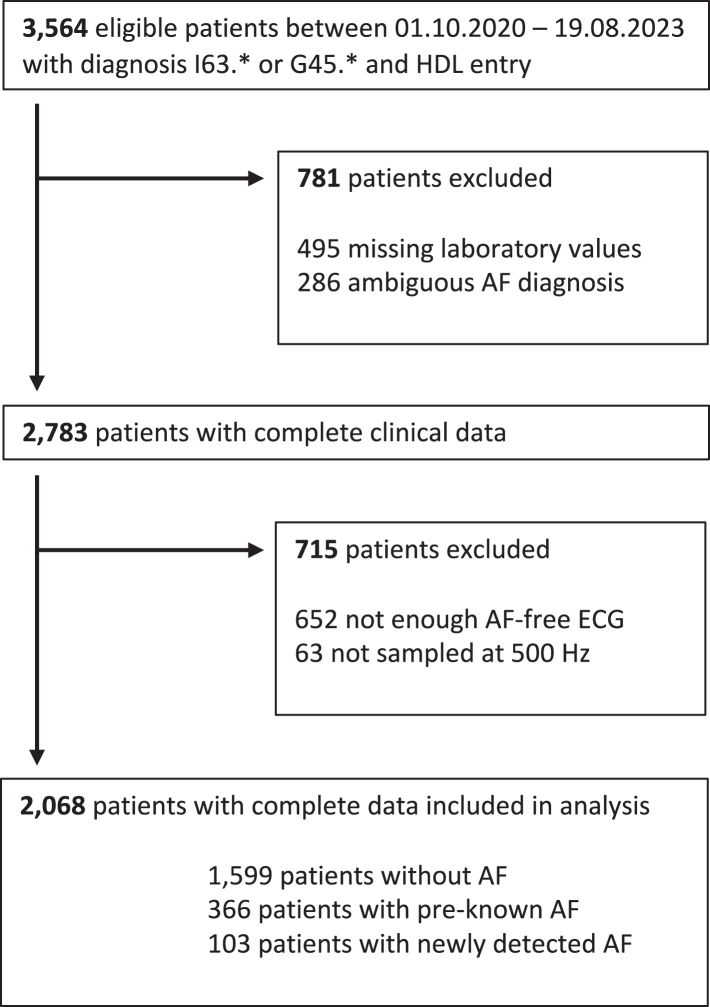
**Flow chart of patient selection.** HDL: Health Data Lake; AF: Atrial fibrillation.

AF was diagnosed by treating physicians if it was detected during hospital stay for the index stroke or when documented in the patients' health record before. The AF diagnosis was retrieved for the purpose of our study from the mandatory stroke register data. Laboratory data and relevant concomitant diagnoses were extracted from the HDL. Patients with incomplete dataset were excluded from further analysis (Fig. 2).

#### Internal validation dataset

The internal validation dataset set consisted of patients treated at the Charité between August 20, 2023 and December 30, 2023, included under the same conditions as those of the derivation dataset.

#### External validation dataset

The external validation dataset originates from the intervention group of the prospective, multi-centre MonDAFIS study (NCT02204267).^24^ In this trial, patients with acute ischaemic stroke or transient ischaemic attack were randomly assigned to usual diagnostic procedures (control group) or additional Holter-ECG recording for up to seven days while in hospital (intervention group). CEM-data from the intervention group was manually labelled by technicians under supervision of a cardiologist. AF was defined as an arrhythmic event lasting for at least 30 s and qualified as AF by the physician.

### Data features and pre-processing

#### Clinical features

The selected clinical features were largely based on previous research identifying them as relevant risk factors associated with AF post-stroke.^14^^,^^22^ We included age, sex, and the National Institute of Health Stroke Scale (NIHSS) scores at admission, the modified Rankin Scale (mRS) scores at admission, binary indicators for the presence of ipsilateral >50% internal carotid stenosis, hypertension, diabetes, heart failure, coronary heart disease, logarithmically transformed troponin levels, and glomerular filtration rate (GFR). NT-pro-BNP-levels and echocardiography data were available only for a minority of patients and were therefore not used.

#### CEM data pre-processing and AF removal

To analyse the CEM data, we used both the raw ECG signal and derived heart rate and HRV metrics from beat-to-beat intervals detected by the proprietary Philips algorithm. In both approaches, patients were included in the analysis only if at least 3 h of CEM data were available during both core daytime (09:00–18:00) and core nighttime (21:00–06:00) periods. Data segments identified as AF by the algorithm were excluded. To ensure comprehensive removal of AF-related activity, an additional buffer of 2.5 min was excluded before and after each identified AF segment.

#### Raw CEM features

Raw CEM data were divided into non-overlapping 5.12-s segments and processed using a Butterworth bandpass filter with a passband of 0.5–50 Hz. Segments were excluded if they contained fewer than three heartbeats or included beats labelled by the Philips system as anything other than normal heartbeat, supraventricular, or ventricular extrasystoles. From the remaining valid segments, 80 segments of 5.12 s each were randomly selected per patient for further analysis.

#### HRV features

HRV features were calculated based on labelled heartbeats and R–R intervals detected by the proprietary Philips algorithm. Consistent with established protocols, we segmented the data into continuous 5-min intervals. Data segments shorter than 5 min were discarded to avoid contamination from concatenation artefacts. Beats, which were not categorised as either normal heartbeat, supraventricular, or ventricular extrasystole by the Philips system, were entirely removed from the dataset.

To minimise data loss, gaps resulting from the removal of ectopic beats were interpolated using the *fixpeaks* function from the NeuroKit2 toolbox when the gap duration was less than 3 s.^25^ For longer gaps, we merged adjacent 5-min segments occurring before and after the interruption. Following extensive testing, we chose not to detrend the ECG signal prior to analysis, as standard detrending methods adversely affected the reliability of non-linear HRV metrics.^25^^,^^26^ A total of 19 HRV features were computed per 5-min segment using the open-source NeuroKit2 Python package^27^^,^^28^ (Supplementary Table S1). For each HRV metric, we computed the mean across all valid 5-min segments within each hour. These hourly averages served as input for model development.

For the external validation dataset, which contained only raw ECG signals, RR intervals were computed prior to HRV analysis using the NeuroKit2's *ecg_peaks* function.^28^

### Model development

We developed three types of models: An ensemble model, a DNN and a fusion model. For training and evaluation of our models we used a stratified shuffle split cross-validation strategy, parameterised by 5 splits and a 20% test set size, employing a seed for consistency across different models. To avoid potential data leakage between the training set and the test sets, we split the data on the patient level.

#### Deep neural network model

For analysis of the raw CEM data, we employed a DNN adapted from the architecture proposed by Attia et al.,^12^ modified to accommodate fewer input channels using the TensorFlow Keras framework. A detailed description of the network architecture is provided in Supplementary Figure S1. The model was trained for 20 epochs using the Adam optimiser with a learning rate of 0.001, and binary cross-entropy was used as the loss function. To determine the optimal quantity of input data, model performance was evaluated across varying numbers of included 5.12-s segments, ranging from 1 to 80 per patient.

#### Heart rate variability and clinical model

To predict atrial fibrillation based on HRV metrics and/or clinical variables, we used ensemble models compiled by the AutoML library.^29^ Model and hyperparameter tuning were performed using a randomised search within an inner cross-validation loop. The best-performing model parameters were then applied to the test set in the outer validation loop.

#### Fusion model

To evaluate whether incorporating raw ECG data alongside HRV metrics and clinical variables enhances predictive performance, we applied Bayesian model averaging to combine the outputs of different models. Specifically, for each test fold, we computed the weighted average of the DNN predictions (based on raw ECG) and the ensemble model predictions (based on HRV and clinical features) using the following equation:

$$P_{Bayesian}=\frac{p_{model1}\cdot p_{model2}}{p_{model1}\cdot p_{model2}\;+\;\left(1\;-\;p_{model1}\right)\cdot \left(1\;-\;p_{model2}\right)}$$

### Statistics

The t-test was used for continuous variables that followed a normal distribution, while the Mann–Whitney U test was applied to non-normally distributed continuous variables. Normality was assessed using the Shapiro–Wilk test. Categorical variables were analysed using the chi-squared test.

Model performance was primarily evaluated using the mean area under the receiver operating characteristic curve (ROC-AUC). On the derivation dataset, ROC-AUC values were assessed using 5-fold cross-validation. Mean ROC-AUC scores across the five folds were reported alongside 95% confidence intervals, calculated using 200-fold bootstrapping per split. To evaluate the statistical significance of performance differences, we applied the DeLong test to each fold.^30^^,^^31^ The resulting p-values were aggregated using Fisher's method [32] to obtain an overall significance estimate.^32^

For the internal and external validation datasets, ROC-AUC values were calculated from a single evaluation run. Corresponding 95% confidence intervals were calculated based on 1000-fold bootstrapping. The DeLong test was used to assess the significance of ROC-AUC differences between models. Additionally, for these out-of-sample tests, sensitivity, specificity, positive predictive value (PPV), and negative predictive value (NPV) were computed at a predefined threshold corresponding to 90% specificity—determined from the derivation dataset. This threshold was selected to reflect the clinical priority of high specificity for effective population enrichment for enhanced screening. Statistical comparison of these secondary metrics was performed using paired t-tests based on 1000-fold bootstrapping, with final p-value aggregation again conducted via Fisher's method.

### Explainability

We conducted comprehensive analyses using SHapley Additive exPlanations (SHAP) to assess feature importance in raw CEM data analysis and multimodal models. To evaluate the DNN's predictive capability, we additionally compared its predictions based on randomly selected CEM segments to predictions based on human labelling.

#### SHAP analysis

We employed SHAP analysis to evaluate the contribution of CEM morphology, HRV metrics, and clinical features to AF prediction.^33^ For the DNN, SHAP was used to interpret the predictive relevance of individual time points within the raw CEM segments. Specifically, we saved the best-performing model from the five training folds. For the selected model, a Deep Explainer object was initialised using 100 patient IDs drawn from the corresponding training set. SHAP values were then computed for the respective test set.

For the models based on HRV and clinical variables, SHAP values were calculated for each fold across all cross-validation repetitions. This allowed us to assess the relative importance of individual HRV and clinical features in contributing to the model's predictions.

#### Human labelling

Additionally, to assess whether the DNN—which presumably leverages CEM morphology in its predictions—could predict AF even in the absence of brief arrhythmic events detectable by human observers, we manually labelled 1000 randomly selected CEM segments from 100 randomly chosen patients with a confirmed AF diagnosis. Importantly, all segments previously identified as AF by the Philips system were excluded prior to this analysis. We then compared the DNN's predictions on CEM data from AF patients with and without visually apparent arrhythmic activity.

### Benchmarking against AS5F score

Several clinical scores of varying complexities have been proposed to predict AF after stroke. The AS5F score^34^ is an externally validated, clinical score comprising only age and NIHSS at admission that repeatedly performed well when compared to other broadly accepted clinical scores.^21^ In a comparative study it outperformed C2HEST, CHADS2, CHA_2_DS_2_-VASc, HATCH, HAVOC, and Re-CHARGE-AF score.^22^ When tested on our derivation dataset, the AS5F performed better than the CHA_2_DS_2_-VASc and comparable to the CHASE-LESS score (Supplementary Figure S2).

To ensure coherent benchmarking in our study, we report the AS5F not only for our external validation dataset but also for our derivation and internal validation dataset, including subsets of our data containing patients with pre-known AF. Comparisons between a model specifically developed and trained on our dataset and previously established scores derived from other datasets inherently favour the newly developed model. Furthermore, the AS5F score was specifically developed to predict previously unknown atrial fibrillation and may exhibit lower performance in the sub-analyses including patients with pre-known AF. Respective analyses of our derivation and internal validation dataset therefore must be interpreted with caution.

### Role of funders

The funders had no role in the study design, data collection, data analysis, interpretation of results, or writing of the report.

## Results

### Model development and comparative testing: multimodality improves performance

We identified 3564 eligible patients matching our diagnosis criteria. Overall, 1496 patients were excluded from further analysis due to missing laboratory values (n = 495), ambiguous information regarding the presence of AF (e.g., no respective diagnosis, but documented AF-alerts in the monitoring data, n = 286), lack of AF-free CEM data (n = 652 patients), CEM not sampled at 500 Hz (n = 63) (Fig. 2). For our primary analysis, we excluded 366 patients with pre-known AF. Out of the remaining 1702 patients (46.8% females, mean age 71.4 years), 103 (6.1%) were newly diagnosed with AF during the index hospital stay (Table 1). Baseline characteristics for the entire dataset including those with pre-known AF are provided in the Supplementary Materials (Supplementary Table S2).

**Table 1:** Baseline characteristics of the derivation dataset.

	All	NDAF	No AF	p-value
Patients	1702	103	1599	
Age, mean (SD)	71.4 (13.3)	79.2 (9.0)	70.9 (13.4)	<0.001^a^
Age, median (IQR)	74.0 (63.0–81.0)	80.0 (73.0–85.0)	73.0 (62.0–81.0)	<0.001^a^
Females, no. (%)	797 (46.8)	54 (52.4)	743 (46.5)	0.283^b^
Males, no. (%)	905 (53.2)	49 (47.6)	856 (53.5)	0.283^b^
AIS, no. (%)	1196 (70.3)	95 (92.2)	1101 (68.9)	<0.001^b^
TIA, no. (%)	506 (29.7)	8 (7.8)	498 (31.1)	<0.001^b^
NIHSS, mean (SD)	3.2 (4.8)	6.7 (6.6)	2.9 (4.5)	<0.001^a^
NIHSS, median (IQR)	1.0 (0.0–4.0)	4.0 (1.5–11.5)	1.0 (0.0–4.0)	<0.001^a^
mRS admission, mean (SD)	1.8 (1.6)	2.9 (1.6)	1.7 (1.6)	<0.001^a^
mRS admission, median (IQR)	1.0 (0.0–3.0)	3.0 (2.0–5.0)	1.0 (0.0–3.0)	<0.001^a^
mRS release, mean (SD)	1.3 (1.5)	2.1 (1.7)	1.2 (1.5)	<0.001^a^
mRS release, median (IQR)	1.0 (0.0–2.0)	2.0 (1.0–3.0)	1.0 (0.0–2.0)	<0.001^a^
Ipsilateral ICA-stenosis > 50%, no. (%)	105 (6.2)	5 (4.9)	100 (6.3)	0.718^b^
Hypertension, no. (%)	1265 (74.3)	79 (76.7)	1186 (74.2)	0.651^b^
Diabetes, no. (%)	373 (21.9)	26 (25.2)	347 (21.7)	0.472^b^
Heart failure, no. (%)	149 (8.8)	16 (15.5)	133 (8.3)	<0.05^b^
Coronary artery disease (CAD), no. (%)	314 (18.4)	16 (15.5)	298 (18.6)	0.512^b^
Time on monitor^c^ [h], mean (SD)	46.5 (16.8)	56.4 (15.0)	45.9 (16.7)	<0.001^a^
Effective monitoring time^d^ [h], mean (SD)	37.5 (14.5)	44.7 (13.6)	37.1 (14.4)	<0.001^a^
AS5F, mean (SD)	65.4 (11.4)	73.9 (9.2)	64.9 (11.4)	<0.001^a^

NDAF: Newly detected AF; AF: Atrial fibrillation; AIS: Acute ischaemic stroke; TIA: Transient ischaemic attack; NIHSS: National Institute of Health Stroke Scale; mRS: Modified Rankin Scale; ICA: Internal carotid artery; NA: Not applicable.

Information on race/ethnicity was not available. The respective baseline characteristics table for the dataset including all AF patients (newly detected and pre-known) can be found in the Supplementary Materials (Supplementary Table S2). A sex-disaggregated baseline characteristics table is also available (Supplementary Table S3).

a Mann–Whitney-U-test.

b Chi-squared test.

c Time from first to last recorded heartbeat.

d Coherent monitor signal minus time windows in which no useable signal was recorded.

**Fig. 3:**
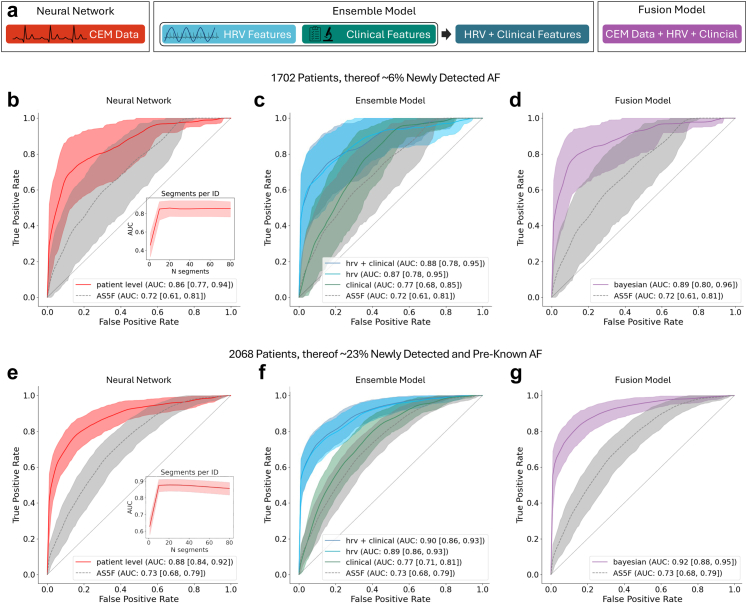
**Results of comparative testing of different models.** a) Schematic depiction of the different classes of features and models. b, c, d) ROC curves of DNN, ensemble and fusion models for patients without AF vs. newly detected AF (1702 patients, thereof 1599 without and 103 with newly detected AF). e, f, g) ROC curves for patients without AF vs. newly detected AF and pre-known AF (2068 patients, thereof 1599 without AF, 103 with newly detected AF and 366 with pre-known AF). Inset Figure 3b and e: Inclusion of more ECG segments increased performance up to 20 segments. All curves are plotted with 95% confidence intervals across folds. ROC-AUCs and respective 95% confidence intervals are provided in the legends. ROC: Receiver operating characteristic; DNN: Deep neural network; CEM: Continuous electrocardiogram monitoring; HRV: Heart rate variability; AF: Atrial fibrillation; AUC: Area under the curve.

The AS5F score achieved a ROC-AUC of 0.72 [95% Confidence Interval (CI): 0.61–0.81]. On the same dataset, an ensemble ML model utilising multimodal clinical data—including baseline characteristics, concomitant diagnoses, and laboratory values—achieved a ROC-AUC of 0.77 [95% CI: 0.68–0.85] (Fig. 3c). As direct comparisons inherently favour models specifically developed and trained on our dataset over previously established scores derived from other datasets, we refrained from calculating p-values to avoid misleading interpretations.

The morphologic properties of ECG in sinus rhythm contain information about underlying atrial disease and may thus inform prediction models. Therefore, we next trained and tested a DNN on 5.12-s CEM segments. We observed a general trend where the inclusion of more segments increased performance up to 20 segments (Fig. 3b inset). A DNN utilising 20 segments or more significantly outperformed the clinical ML model (ROC-AUC 0.86 [0.77, 0.924], p = 4.68e-03 [DeLong's test, Fisher's method]; Fig. 3b).

Long-term patterns in CEM signals captured by HRV metrics may contain complementary information to that represented by P-wave and QRS complex morphology. HRV-based models have consequently been used to identify patients at high risk for AF.^17^^,^^18^ Here, an ensemble ML model using HRV metrics as input achieved a ROC-AUC of 0.87 [0.78, 0.95], significantly outperforming the clinical ML model (p = 3.45e-04 [DeLong's test, Fisher's method]) but not the DNN (p = 4.28e-01 [DeLong's test, Fisher's method]). We next evaluated whether including multimodal clinical variables in addition to HRV would further improve predictions. This approach achieved a slightly higher ROC-AUC of 0.88 [0.78, 0.95] than HRV alone or the DNN (p = 2.06e-01 and p = 6.44e-01, respectively [DeLong's test, Fisher's method]).

Considering that DNNs assess morphological features from raw CEM data which may contain complementary information to long-term trends in HRV, we next assessed performance of a Bayesian fusion model combining both DNNs on raw CEM data and ensemble ML models analysing HRV, along with clinical information. This model achieved the highest ROC-AUC of 0.89 [0.80, 0.96] (Fig. 3d), outperforming the DNN (p = 2.92e-04 [DeLong's test, Fisher's method]) but not reaching the level of significance for the ensemble ML models using HRV or HRV and clinical data (p = 3.36e-01 and p = 4.83e-01, respectively [DeLong's test, Fisher's method]).

In an additional analysis that included patients with both newly detected and pre-known AF, the relative performance of the different models remained unchanged, while the overall ROC-AUC values were slightly higher (Fig. 3e–g). The respective data along with additional sensitivity analyses is provided in the Supplementary Materials (Supplementary Figure S3 and Supplementary Results).

### Feature explainability: ECG and HRV are largest contributors to algorithm performance

**Fig. 4:**
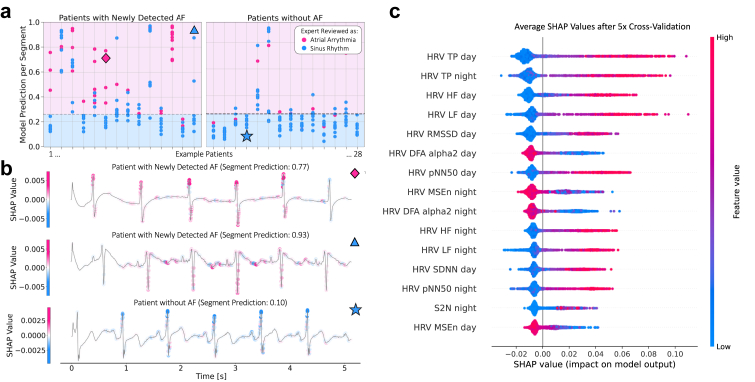
**Explainability analysis.** a) Distribution of predictions generated by the DNN model based on distinct CEM segments for 28 example patients. Each column corresponds to a unique patient and shows the predictions for 10 ECG segments (duration 5.12 s each) for this patient. Segments labelled as sinus rhythm by human experts are depicted in blue, while those labelled as potential atrial arrhythmia are shown in pink. The dashed horizontal line shows the 90% specificity threshold. b) SHAP analysis on three raw CEM example segments. Icons in the upper right corner mark the corresponding segments in panel A. Top: segment from a patient with AF diagnosis which was classified as atrial arrhythmia by the human expert and as indicative for underlying AF by the model. Middle: segment from a patient with AF diagnosis which the human expert categorised it as sinus rhythm while the DNN classified it as indicative for underlying atrial fibrillation. Bottom: segment from a patient without AF diagnosis that was categorised as sinus rhythm by the human expert while the model did classify it as not indicative for underlying AF. c) SHAP analysis of the relative predictive performance of individual features applied to the ensemble model (HRV + clinical). DNN: Deep neural network; CEM: Continuous electrocardiogram monitoring; AF: Atrial fibrillation; SHAP: SHhapley Additive exPlanations; HRV: Heart rate variability; TP: Total power; HF: High frequency, LF: Low frequency, RMSSD: Root mean square of successive differences; DFA: Detrended fluctuation analysis; pNN50: Proportion of the number of pairs of successive normal heartbeats (NN) that differ by more than 50 ms divided by the total number of NN; MSE: Multiscale entropy; SDNN: Standard deviation of NN intervals; S2N: Number of supraventricular extrasystoles divided by the number of normal heartbeats. For more detailed information see Supplementary Table S1.

Predictions of the ensemble ML model and the DNN were assessed separately for feature explainability. For the ensemble ML model, HRV-features representative of long-term modulations along with HRV-features indicative of high short-term variability were identified as the most informative features. Conversely, clinical variables such as age, sex, NIHSS, mRS, and concomitant diagnoses had a lower impact on model classification (Fig. 4c).

Analysis of the DNN highlighted the importance of individual ECG segment points in the model's decision-making process. Results of this analysis suggest that the distance between the R-peaks (and thus rhythmicity of the signal) is considered the most informative part by our model along with morphologic properties of the P-wave or, respectively, its absence (Fig. 4b). Furthermore, comparison with hand-labelled CEM data segments indicated that the DNN based its predictions largely on the presence or absence of short arrhythmic events (<30 s) and also—to a lesser extend—on other morphologic features contained in sinus rhythm ECG within the analysed CEM data (Fig. 4a).

### Design and selection of final model: optimised performance for broad applicability

Our cross-validation results suggested that most information was captured by HRV and a few selected clinical variables, and that DNNs added only moderately to the predictive performance. Considering further the demand for high data quality and computing capacities of DNNs, we next aimed to establish a model that required only limited input while providing excellent predictive performance. Specifically, to facilitate automated clinical application in the post-stroke setting we prioritised practicality throughout the optimisation process, emphasising baseline characteristics known at the time of admission over lab values requiring testing or diagnoses confirmed by costly methods. A model utilising only age in addition to HRV data, provided the best trade-off between predictive performance and ease of use. This model achieved a ROC-AUC only slightly lower than that of our most preformant Bayesian fusion model while facilitating broad application due to its lean design (Dataset including newly detected AF only: ROC-AUC for the simplified model: 0.88 [0.79, 0.95]; ROC-AUC for the Bayesian fusion model: 0.89 [0.80, 0.96], p = 4.57e-01 [DeLong's test, Fisher's method]. Dataset including newly detected AF and pre-known AF: ROC-AUC for the simplified model: 0.90 [0.86, 0.93]; ROC-AUC for the Bayesian fusion model: 0.92 [0.88, 0.95], p = 2.69e-03 [DeLong's test, Fisher's method]).

Next, we investigated how reducing the length of CEM data might still yield valid predictions, thereby allowing earlier AF-risk stratification upon stroke unit admission. While patient age is immediately available, HRV features used by the model require calculation from the CEM data, meaning that less CEM data used translates into earlier prediction. We systematically compared models incorporating age and HRV features calculated from 8 h, 4 h, 2 h, 1 h, 30 min, 15 min, and, 5 min of CEM data (Supplementary Figure S4). Ultimately, a model based on only 1 h of CEM data plus age emerged as the final choice, balancing performance, ease of use, and timeliness.

To internally validate our final model (input restricted to age and HRV values calculated on the first hour of CEM data only), we analysed data from a hold-out dataset of 397 patients (46.1% female, mean age 74.0 years). 87 (21.9%) were labelled as having AF (71 with pre-known AF, 16 with AF diagnosed during index hospital stay, Supplementary Table S5). On this dataset the final model performed better than the AS5F-score (pre-known and newly detected AF: ROC-AUC 0.87 [0.81, 0.91] vs. 0.71 [0.65, 0.77], p = 1.39e-10 [DeLong's test]; newly detected AF only: ROC-AUC 0.80 [0.66, 0.92] vs. 0.70 [0.54, 0.85], p = 0.12 [DeLong's test]; Supplementary Figure S5).

### External validation

**Fig. 5:**
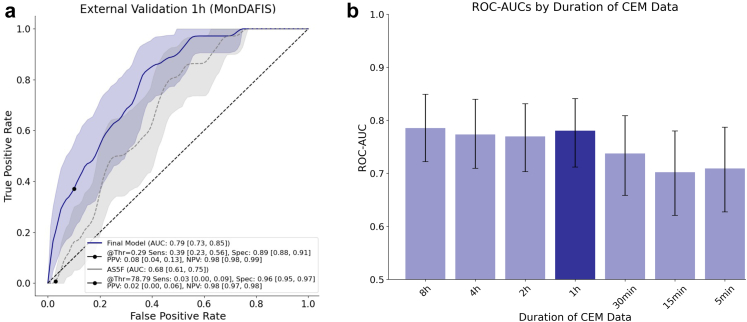
**External validation.** a) Validation on the MonDAFIS-dataset, using only the first hour of CEM data. b) Validation on the MonDAFIS-dataset, incorporating different lengths of CEM data for HRV calculation. p-value for final model vs. AS5F was 4.69e-03 [DeLong's test]. CEM: Continuous electrocardiogram monitoring; AUC: Area under the curve; Thr: Threshold; Sens: Sensitivity; Spec: Specificity; PPV: Positive predictive value; NPV: Negative predictive value.

For external validation under real-world conditions, we applied our final model to the first hour of CEM data from the MonDAFIS dataset.^24^ The external MonDAFIS-dataset contained 1714 patients from the intervention group of the trial of which 195 patients had to be excluded (29 due to missing baseline characteristics, 121 because AF was detected during long term follow up, 33 because AF had been detected by routine monitoring, 7 because AF was detected before or within the first useable hour of Holter-monitoring, 5 due to insufficient quality of the recording). Of the 1519 remaining patients (60.2% females, mean age 65.6 years), 36 (2.37%) were labelled as having AF (Supplementary Table S6). On external validation, the final model predicted AF with a ROC-AUC of 0.79 [0.73, 0.85], outperforming the AS5F-score (ROC-AUC of 0.68 [0.61, 0.75], p = 4.69e-03 [DeLong's test]; Fig. 5a). At the predefined threshold (90% specificity in the derivation dataset), the sensitivity was 0.39 [0.23, 0.56] (AS5F: 0.03 [0.00, 0.09], p < mp (machine precision) [paired t-test, Fisher's method]), positive predictive value was 0.08 [0.04, 0.13] (AS5F: 0.02 [0.00,0.06], p < mp [paired t-test, Fisher's method]), and negative predictive value was 0.98 [0.98, 0.99] (AS5F: 0.98 [0.97, 0.98], p = 4.29e-314 [paired t-test, Fisher's method]). 164 of 1519 patient (10.8%) were classified as being at high risk for underlying AF by our model. Again, 1 h of CEM data provided a good balance between performance, limited data requirements and timely predictions (Fig. 5b). Additional benchmarking of our model demonstrated favourable performance also against other established clinical scores (Supplementary Figure S6). In an additional sensitivity analysis excluding all manually labelled short atrial runs, our model still achieved a ROC-AUC of 0.79 [0.72, 0.85]. This supports findings from our explainability analysis, which suggest that HRV captures predictive information beyond the presence or absence of brief arrhythmic events (Supplementay Figure S7).

## Discussion

In this study, we aimed to develop a machine-learning model to predict AF in patients with acute ischaemic stroke or transient ischaemic attack. We retrospectively analysed data from 2068 patients treated at our institution, trained several models using different architectures and input features, and compared their performance to identify the most informative predictors. We then simplified the model for practical clinical use, externally validated this final version in a cohort of 1519 patients, and benchmarked it against established clinical risk scores.

Our main findings are: 1. Metrics assessing ECG rhythmicity—particularly HRV metrics —appear to be at least as informative for detecting underlying AF as ECG morphology or conventional clinical variables. 2. A streamlined machine-learning model, using only patient age and HRV metrics derived from 1 h of CEM, can predict AF for up to seven days after stroke with a ROC-AUC of 0.79, outperforming the validated AS5F risk score upon external validation.

So far, no systematic, comparative evaluation of model architectures and data sources for optimal prediction of AF in the context of stroke had been done, resulting in only few published ECG-based models. Two studies reported on testing AI-models developed on ECG-data from patients without stroke on smaller datasets from patients with ESUS and implantable cardiac monitors (ICM),^35^^,^^36^ achieving ROC-AUCs of 0.81 and 0.83 respectively. However, different definitions of AF used in these trials (>1 h and >5 min respectively) make direct comparison difficult. One trial evaluated the AI-model proposed by Attia et al. on a cohort of patients with ischaemic stroke, including 226 patients with ESUS and ICM. OR for AF detection was 5.5 for “high risk” patients as determined by the AI. A ROC-AUC was not reported.^37^ In a similar study, a previously published model combining 12-lead-ECG-analysis with the CHARGE-AF-score correctly classified strokes as cardioembolic with a ROC-AUC of 0.76. While this model also stratified risk for subsequent AF detection, a ROC-AUC for AF prediction specifically was not reported.^38^ None of these studies provided an in-depth assessment of different model architectures or input features.

Despite its clinical relevance, short-term prediction of AF after stroke remains underexplored. One study conducted across two Taiwanese hospitals developed a model to identify patients with stroke and underlying AF diagnoses based on ECG in sinus rhythm with a ROC-AUC of 0.69. Notably, the analysed cohort included patients with pre-known AF.^39^ Adami et al. evaluated a commercial CEM-analysis-software on a single-centre dataset of 200 patients, using a dichotomised risk stratification.^40^ In this study, the diagnosis of AF was more common among patients classified as “high risk” compared to patients classified as “low risk” (38.5% vs. 0.9%). In a study published by Gröschel et al., a combination of the established AS5F-score and the same commercial algorithm achieved a ROC-AUC of 0.79 when used on a dataset of Holter ECGs from the IDEAS-trial, providing a predictive power similar to the one reported here.^41^ However, this approach lacks external validation and a publicly accessible algorithm.

Here, we systematically evaluated various input features and model combinations with respect to AF prediction. We thereby identified various HRV measures as main drivers of our models' predictions. Derived from RR-time series, these metrics represent short-term irregularities and a lack of stable long-term patterns. Many other clinical variables known to be associated with AF contributed only little to the performance of our models. In line with recent publications, inclusion of DNN-enabled analysis of raw CEM data did improve our models' predictive performance.^42^ However, the increase was only moderate. SHAP analysis and comparative analysis of visually inspected CEM-segments revealed that the signal's rhythmicity rather than P-wave-morphology appears to be the primary driver of the network's predictions. This information might already be captured by HRV analysis. Furthermore, our findings indicate that analysis remains reliable even for short periods of 1 h or less. Interestingly, a trial that used P-wave-morphology-alterations as a selection criterion to justify anticoagulation in patients with cryptogenic stroke failed to demonstrate a clinical benefit of this approach.^5^ In light of our findings, we hypothesise that alterations of HRV and the presence of short arrhythmic events rather than morphologic changes of the ECG might be predictive for underlying AF in patients with ischaemic stroke. Supraventricular extrasystoles and atrial runs might play a crucial role.^43^^,^^44^

We propose a lean ML model to predict AF in patients with acute ischaemic stroke or transient ischaemic attack in the absence of apparent AF episodes using only HRV and age as input. This model significantly outperforms the validated AS5F risk score when tested on a large, hand-curated external Holter-ECG dataset. At a pre-defined specificity threshold of 90% PPV for AF-detection within 7 days was 0.08 while the NPV was as high as 0.98. This reasonable PPV at high NPV may allow for a rational and cost-effective screening strategy. The model presented here thus overcomes several shortcomings of previously reported studies: It was developed and validated on large stroke unit datasets. It was externally validated on a large independent, hand-curated, dataset and detailed model introspection ensured explainability. It focusses on clinically relevant, short-term AF prediction, addressing concerns about the significance of AF-episodes detected long after the index event. Importantly, it was tailored to broad clinical application. Since it uses monitoring data recorded automatically and the patient's age, no further human input is needed.

Our study has several limitations. While our data warehouse approach allows for the inclusion of large datasets with multiple variables, it poses challenges for quality control. As our study focused on CEM data from a real-world clinical setting, raw ECG traces were consistently available only for lead II. As a result, the performance of our DNN model can only be evaluated with respect to this lead. However, this limitation does not affect analyses based on HRV, which served as the basis for our final predictive model. Average effective CEM time within the first 72 h after admission in our derivation and internal validation cohort was only 38.3 h, reflecting real world conditions in a large German stroke centre. The classification of individuals into the AF group (true positives) was based on diagnoses coded in quality control registers, which may not always be accurate. Some cases of AF may have been missed by treating physicians. Furthermore, our training data is limited to the hospital stay, and episodes of AF detected after discharge were not considered–leading to an underestimation of AF prevalence in our dataset. Identification of AF-episodes for data pre-processing relied on the proprietary Phillips algorithm. As previously demonstrated, accuracy of this algorithm is limited, leading to potential contamination of the remaining monitoring data.^45^ Additionally, the composition of our training and internal validation cohort does not fully reflect that of patients with cryptogenic stroke, as most patients with AF in this cohort would likely have been identified by conventional monitoring methods as well. Within the frame of our derivation-validation design, the derivation (and internal validation) dataset served to develop and compare different types of prediction models. ROC-AUCs reported here must be interpreted with caution.

Further, due to varying definitions of AF across studies, differences in study populations, diverse gold standards for validation (Holter-ECG, ICM, electronic health records), the lack of external validation in some cases, and our focus on usability rather than predictive power alone, direct comparisons between our model's performance and the performance of other published prediction models are challenging. Despite these limitations, however, our final model performed well when applied to expert-labelled external validation data. The MonDAFIS-dataset meets two critical validation requirements: It reflects the real-world situation for which our algorithm was developed, and it provides contamination-free sinus rhythm CEM data. Results from validation runs on this external dataset therefore can be considered generalisable and representative of what can be achieved under real world conditions. Of note, all patients were recruited in German stroke centres and had to be able to provide informed consent, which may have introduced a selection bias. Nevertheless, the baseline characteristics of our cohort align closely with those reported in recent stroke trials.

Our model is validated for predicting AF only up to seven days post-stroke, and the validation dataset did not include patients who had AF detected through ICM. However, in the context of stroke, the clinical significance of asymptomatic, low-burden AF detected long after the initial event—particularly by highly sensitive methods such as ICM—remains uncertain. Recent studies in non-stroke populations have reported mixed findings regarding the utility of ICM for stroke prevention.^9^^,^^10^ Consequently, there is insufficient evidence to support routine long-term ICM screening in patients with ischaemic stroke, and in most countries, such interventions are not reimbursed. Thus, ICM screening has not become widespread clinical practice, even in industrialised nations. Therefore, we specifically aimed to develop a model for short-term AF prediction, as this is highly relevant for daily clinical practice.

The presented model may serve as a robust tool to identify patients at increased risk for first detection of AF. Such a tool may support more targeted monitoring strategies. Identifying previously undetected paroxysmal AF is critical, as it significantly influences patient management by shifting secondary prevention strategies from antiplatelet therapy to anticoagulation, thus substantially reducing the risk of recurrent strokes. Current European guidelines recommend screening all patients after acute ischaemic stroke by CEM or Holter-ECG for at least 48 h and additional prolonged monitoring after discharge for those with cryptogenic stroke.^4^ However, with rising stroke incidence and increasing economic constraints, adherence to this standard is challenging in clinical practice and burdensome for patients. An individualised, automated risk assessment approach, such as developed here using only 1 h of CEM, might enable shorter monitoring periods for patients with very low AF risk, freeing resources to extend monitoring durations for those at higher risk. In addition, this approach may inform recommendations about intensified, prolonged cardiac monitoring after discharge. Such tailored monitoring strategies could ultimately increase AF detection rates, optimise secondary prevention, improve long-term patient outcomes, and simultaneously reduce healthcare costs.

Broad clinical adoption of our model will require additional, independent external validation. Our findings may also motivate prospective trials including the implementation of our model in routine workflows after stroke. Patients would be automatically and continuously assessed during their stroke workup monitoring to estimate AF risk. A corresponding trial at our centre is currently in preparation. Beyond optimising monitoring strategies, a prospectively validated model may help identify high-risk patients suitable for future trials evaluating oral anticoagulation in subsets of patients with ischaemic stroke currently classified as having cryptogenic stroke.

## Contributors

C.M., M.S., L.K., and A.N. contributed to the conception and design of the study. A.N. and M.K. contributed to the acquisition of data. M.S., L.K., and C.M. drafted the manuscript. M.S., L.K., and A.N. prepared the figures and tables. L.K. and A.N. pre-processed and analysed the data and applied all machine learning methods. C.M., M.S., L.K., and A.N. have accessed and verified the underlying data. All authors contributed to the revision and editing of the manuscript, reviewed the final version, and approved it for submission.

## Data sharing statement

Code for the analysis is available under https://gitlab.com/computational-neurologie/af_prediction_stroke_unit.git. The dataset used for model derivation and internal validation is not publicly available, as no patient consent or ethical approval was obtained for open data sharing. Investigators interested in additional analyses may contact the corresponding author; data access will be considered on a collaborative basis in accordance with institutional guidelines at Charité – Universitätsmedizin Berlin. The dataset used for external validation (MonDAFIS study, NCT02204267) is available from the study investigators upon reasonable request, on a collaborative basis and with investigator support.

## Declaration of interests

CHN received speaker and/or consultation fees from Alexion, Astra Zeneca, Bristol-Myers Squibb, Novartis, Pfizer Pharma, and Bayer, all outside the submitted work. JFS received research grants from the German Heart Foundation and speaker/consultation honoraria from Medtronic, Astra Zeneca, Bayer, and Bristol-Myers Squibb, outside the submitted work. MGK received a grant from the German Heart Foundation. AM received research grants from the German Research Foundation and the Leducq Foundation. KGH reports speaker's honoraria, consulting fees, lecture honoraria and/or study grants from Bayer, AstraZeneca, Pfizer, Bristol-Meyer-Squibb, Daiichi Sankyo, Boehringer Ingelheim, and Novartis. ME reports grants from Bayer and Ipsen and fees paid to the Charité from Amgen, AstraZeneca, Bayer, BMS, Daiichi Sankyo, all outside the submitted work.

The remaining authors have no conflicts of interest or competing interests to declare.

## Appendix A Supplementary data

Supplementary Tables

Caption for Supplementary Figures

Supplementary Results
