# Supplementary figures and images for: Artificial intelligence for prediction of atrial fibrillation in the stroke unit: a retrospective derivation validation cohort study
Source: eBioMedicine. 2025 Aug 5;118:105869. doi: 10.1016/j.ebiom.2025.105869 (PMC12341230; doi:10.1016/j.ebiom.2025.105869)

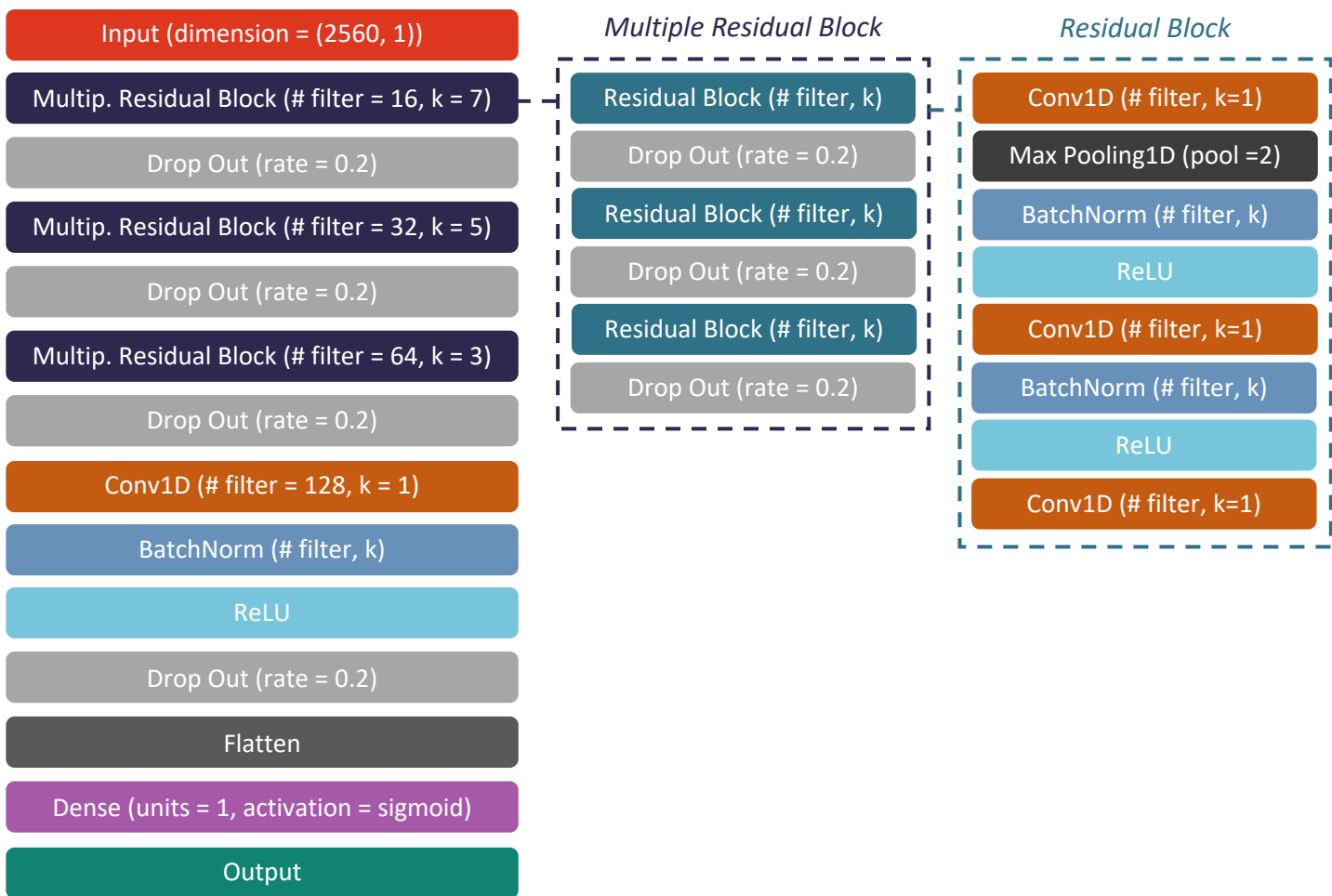

Supplement: Supplementary Figure S1 [file mmc1.pdf]

# Comparative Testing of Established Scores

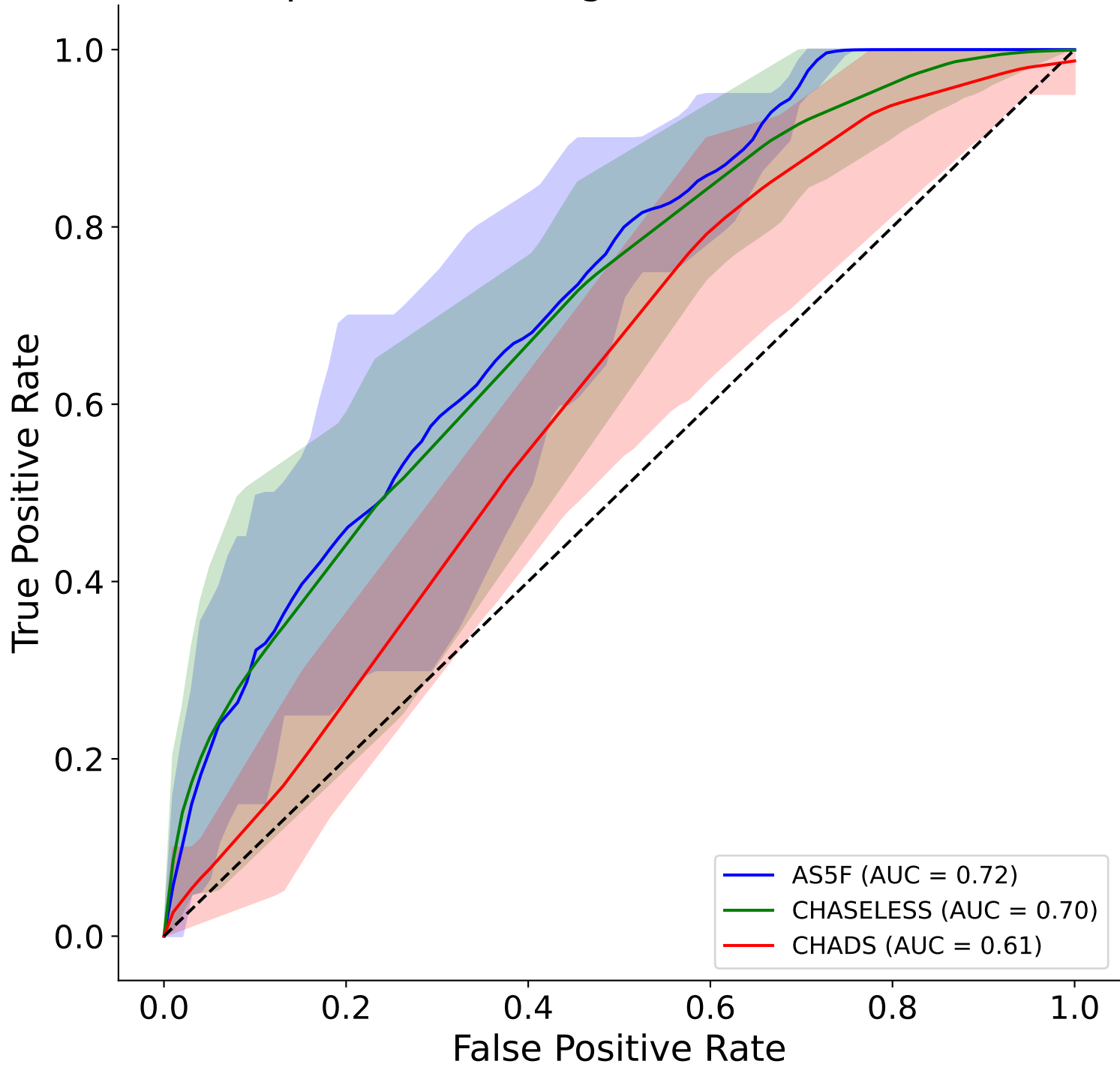

Supplement: Supplementary Figure S2 [file mmc2.pdf]

a

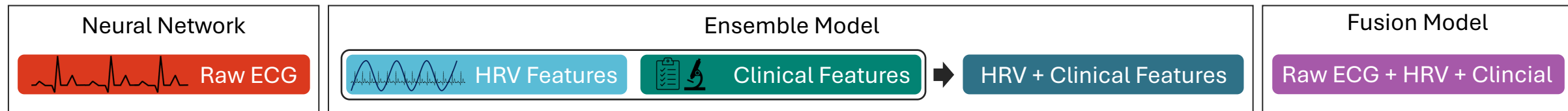

b

ROC Curves - Neural Network

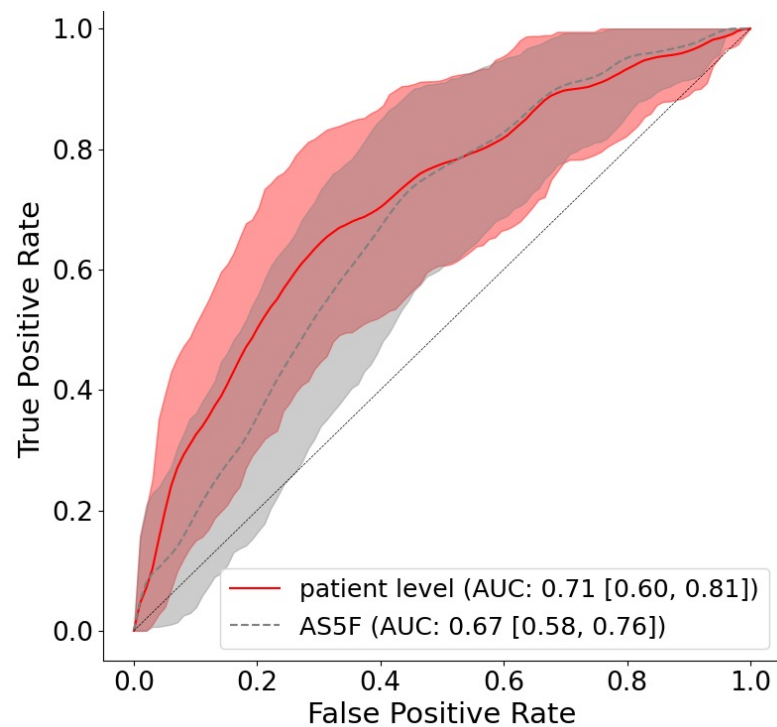

c

ROC Curves - Ensemble Model

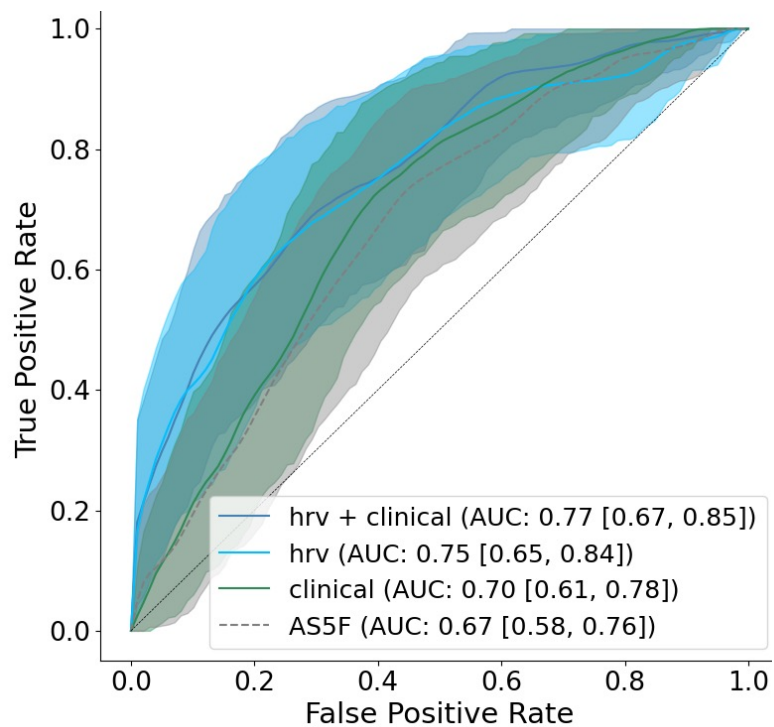

d

ROC Curves - Fusion Model

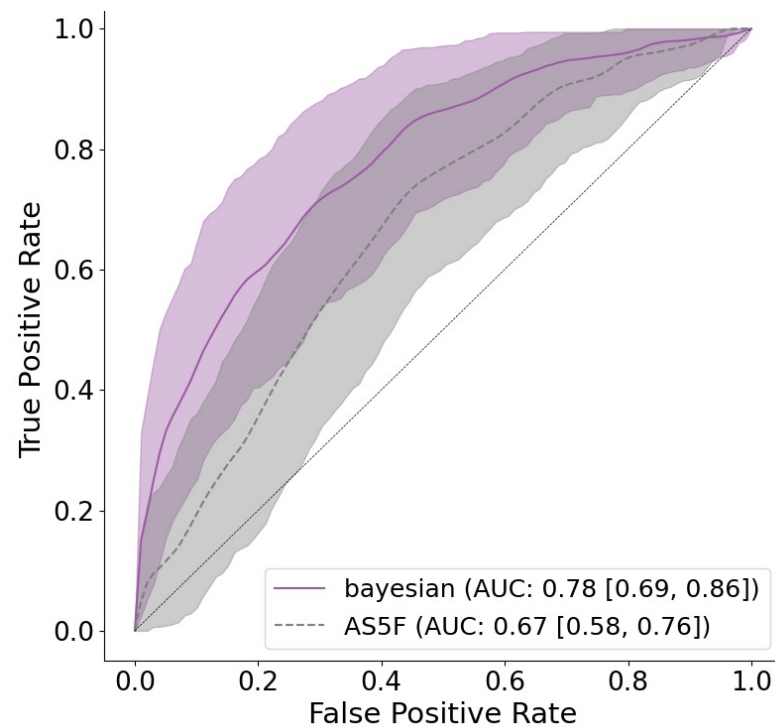

Supplement: Supplementary Figure S3 [file mmc3.pdf]

Simplified Model - ROC-AUCs by Duration of CEM Data

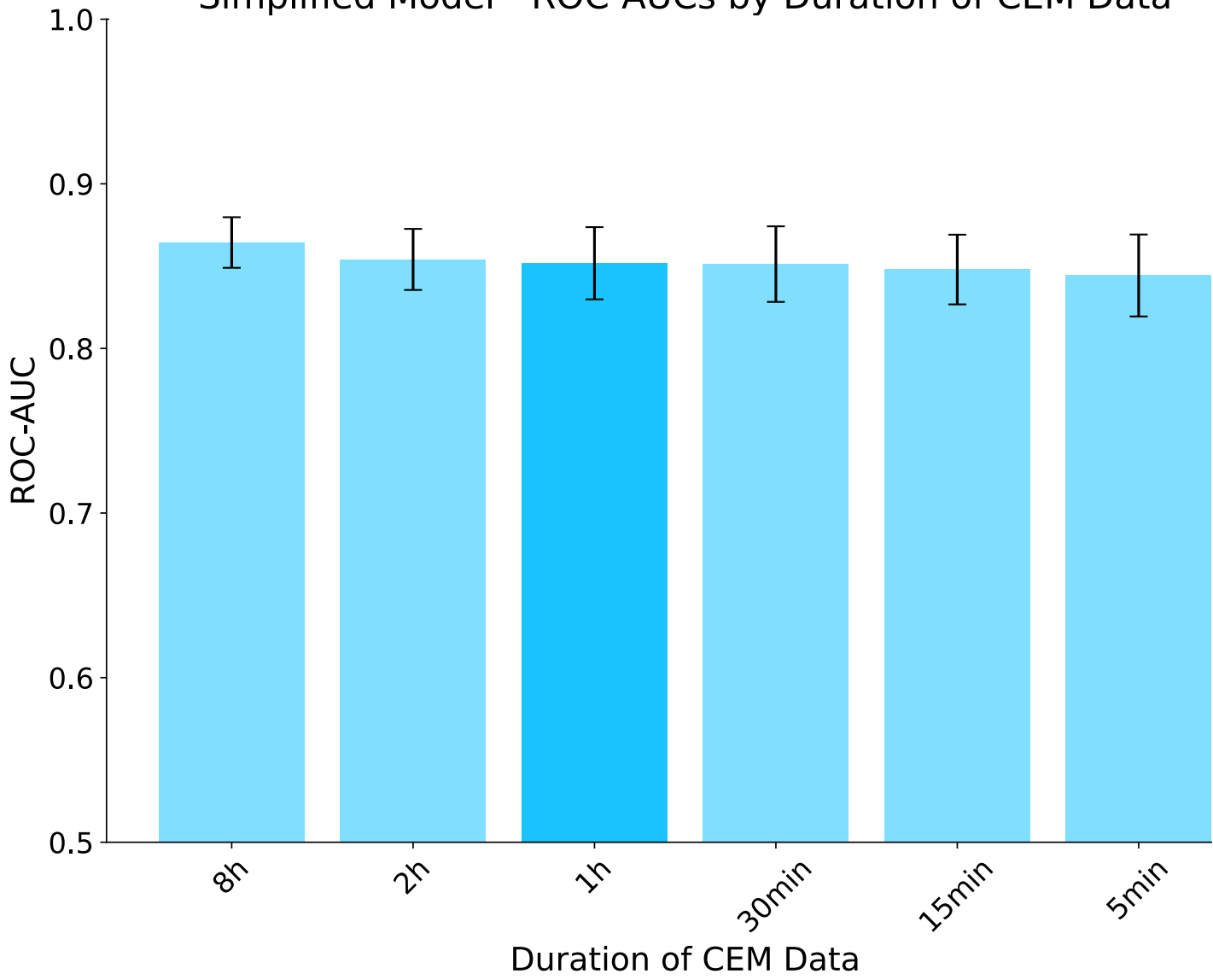

Supplement: Supplementary Figure S4 [file mmc4.pdf]

Internal Validation 1h Newly Detected and Pre-Known AF

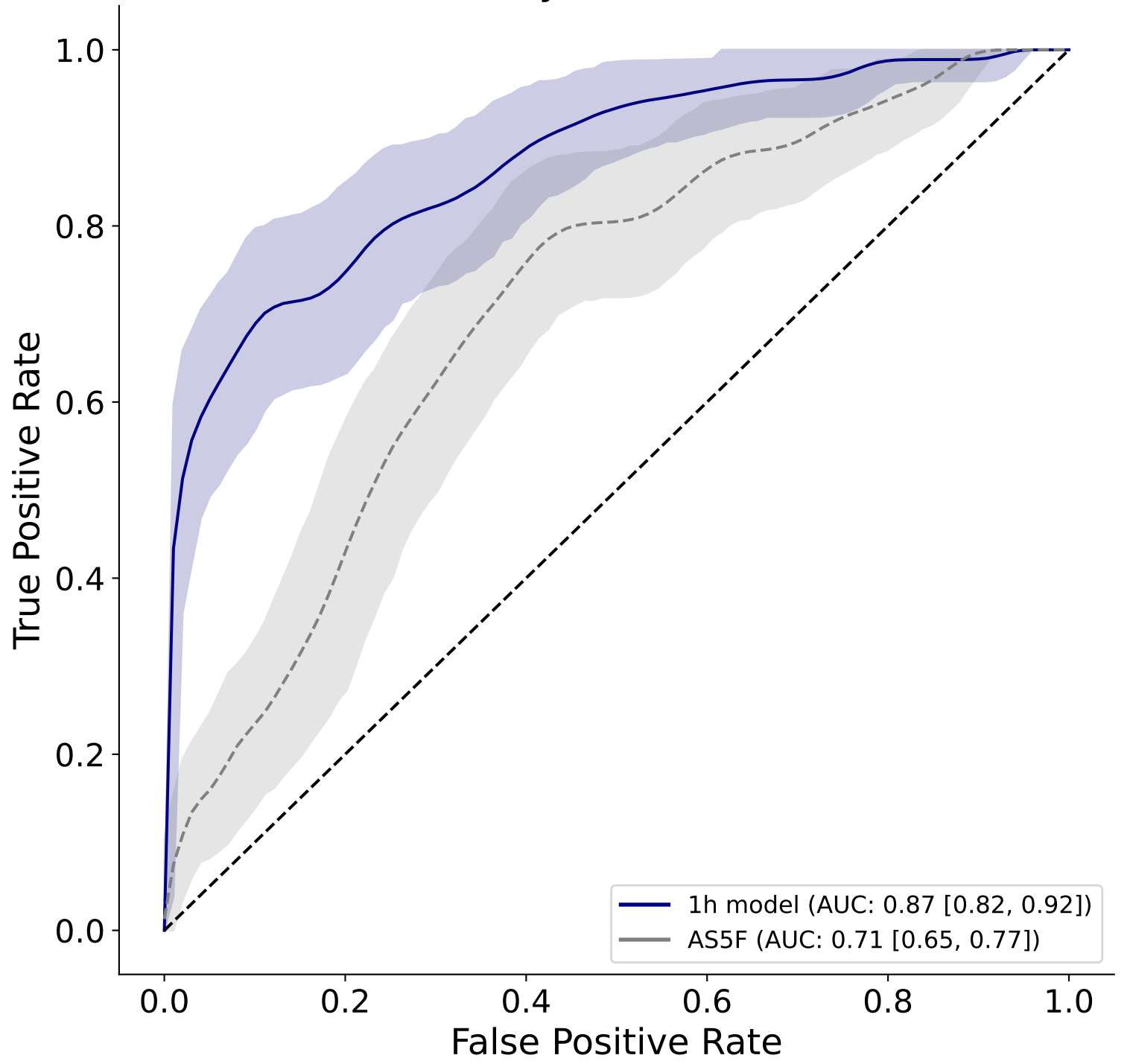

Supplement: Supplementary Figure S5 [file mmc5.pdf]

# Score Performance on External Validation Dataset

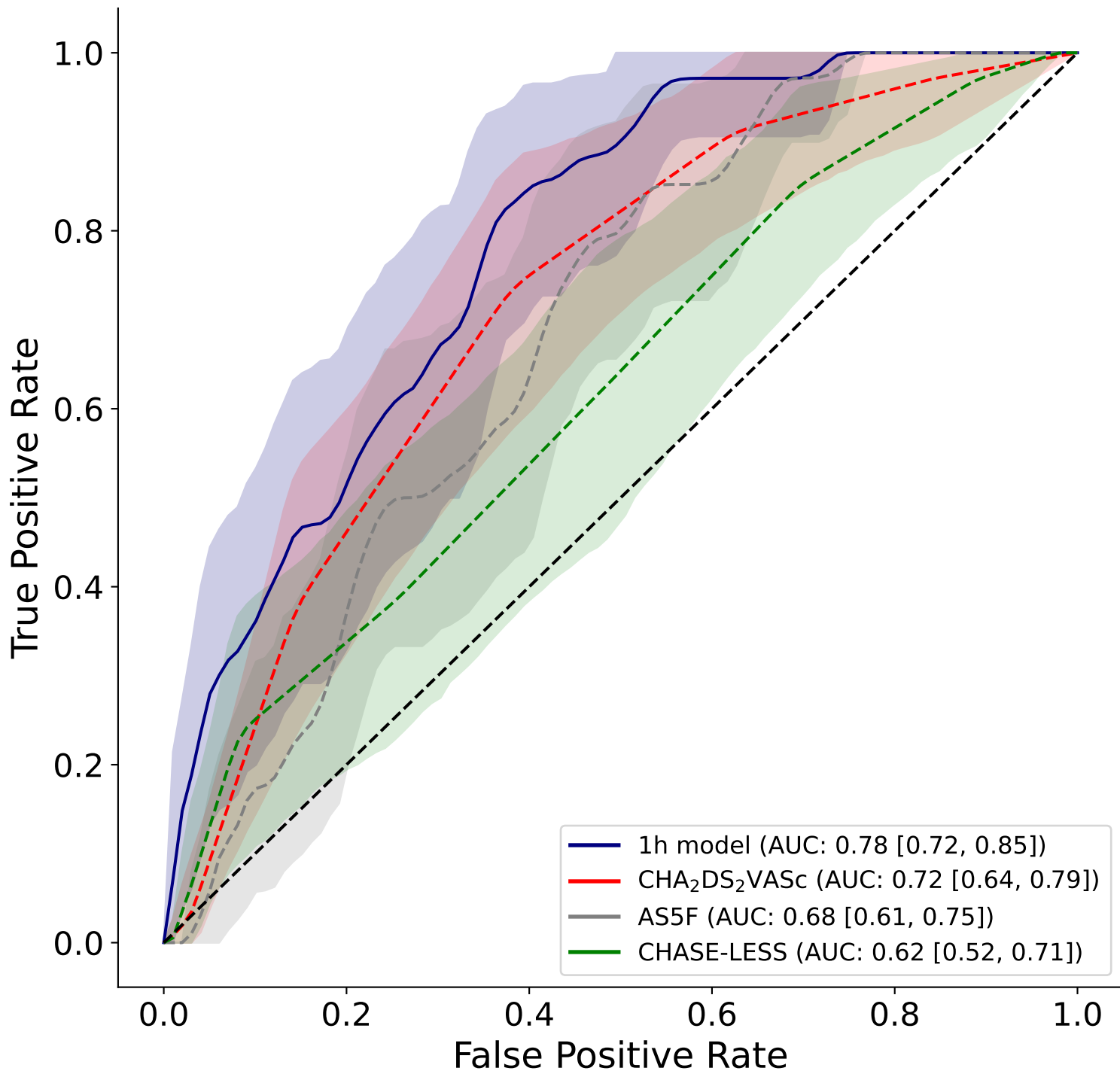

Supplement: Supplementary Figure S6 [file mmc6.pdf]

# External Validation (Atrial Runs Removed)

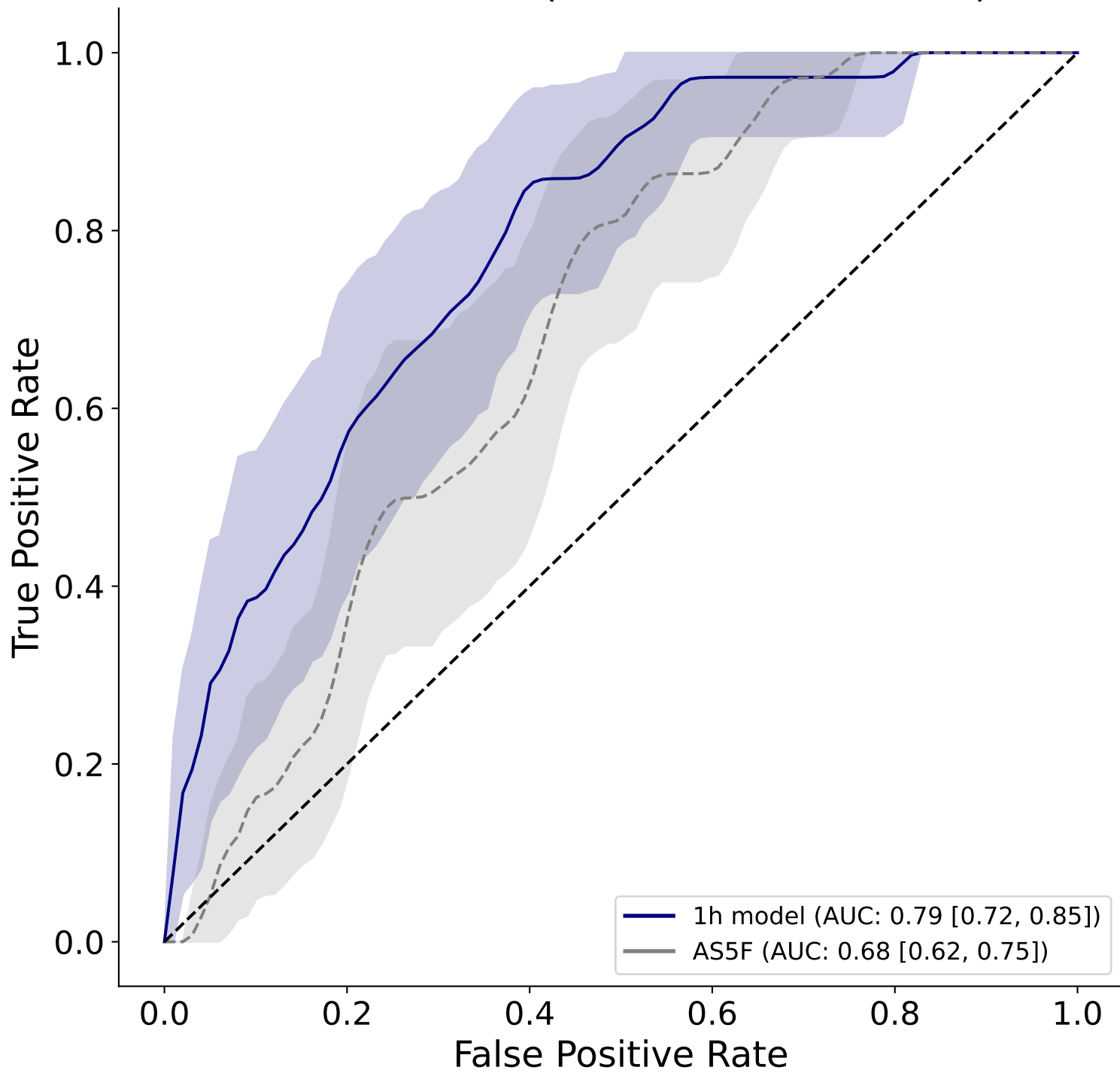

Supplement: Supplementary Figure S7 [file mmc7.pdf]
