# Supplementary material for: Artificial intelligence for prediction of atrial fibrillation in the stroke unit: a retrospective derivation validation cohort study
Source: eBioMedicine. 2025 Aug 5;118:105869. doi: 10.1016/j.ebiom.2025.105869 (PMC12341230; doi:10.1016/j.ebiom.2025.105869)
Supplement: Supplementary Tables [file mmc8.docx]

# Supplementary Tables

## Table S1 Metrics of Heart Rate Variability (HRV)

| Frequency Domain | | |
| --- | --- | --- |
| HF | High-Frequency Power | Power in the frequency band from 0.15 to 0.4 Hz, representing parasympathetic activity |
| LF | Low-Frequency Power | Power in the frequency band from 0.04 to 0.15 Hz, indicative of both sympathetic and parasympathetic modulation |
| VLF | Very Low-Frequency Power | Power in the frequency band from 0.0033 to 0.04 Hz, thought to be mainly dominated by sympathetic activity |
| TP | Total Power | The sum of the power in all frequency bands, a marker of overall HRV |
| Time Domain | | |
| RMSSD | Root mean square of successive differences between normal heartbeats | A measure of short-term variations in heart rate |
| SDNN | Standard deviation of NN (normal to normal heartbeat) intervals | Reflecting overall autonomic nervous system activity |
| PNN50 | Proportion of NN50 divided by the total number of NN intervals | A measure of short-term variations in heart rate and closely related to RMSSD |
| Heart Rate Fragmentation Metrics | | |
| PIP | Percentage of zero-crossing points in the increment time series | A measure of heart rate fragmentation, thought to be representing alterations in Sinus- and AV-node function |
| PAS | Percentage of NN intervals in alternation segments | An alternation segment is a sequence of at least four NN intervals, for which heart rate acceleration changes sign every beat; a measure of heart rate fragmentation |
| Non-Linear Metrics | | |
| FuzzyEn | Fuzzy entropy | A measure of the complexity of RR interval time series |
| MSEn | Multiscale entropy | A metric quantifying the complexity of heart rate dynamics over multiple time scales |
| DFA_alpha1 | Short-term exponent of the detrended fluctuation analysis | A metric assessing the fractal scaling properties of the signal |
| DFA_alpha2 | Long-term exponent of the detrended fluctuation analysis | A metric assessing the fractal scaling properties of the signal |
| Heartrate Measures | | |
| MaxNN | maximum N-N-interval | Represents the lowest heartrate measured |
| MinNN | minimum N-N-interval | Represents the highest heartrate measured |
| MedianNN | median N-N-interval | Represents the median heartrate measured |
| Extrasystoles | | |
| S2N | Supraventricular extrasystoles/normal heartbeat | Ratio of supraventricular extrasystoles to normal heart beats |
| V2N | Ventricular extrasystoles/normal heartbeat | Ratio of ventricular extrasystoles to normal heart beats |
| SV2N | Any extrasystole/normal heartbeat | Ratio of both, supra- and ventricular extrasystoles to normal heart beats |

##

## Table S2 Baseline Characteristics of the Derivation Dataset Including Patients with Pre-Known AF

|  | Total | AF | no AF | p-value |
| --- | --- | --- | --- | --- |
| Patients | 2068 | 469 | 1599 |  |
| Pre-Known AF |  | 366 | 0 |  |
| Newly Detected AF |  | 103 | 0 |  |
| Age, Mean (SD) | 73.1 (13.1) | 80.4 (8.7) | 70.9 (13.4) | < 0.001 |
| Age, Median (IQR) | 76.0 (65.0 - 82.0) | 82.0 (76.0 - 86.0) | 73.0 (62.0 - 81.0) | < 0.001 |
| Females, no. (%) | 983 (47.5) | 240 (51.2) | 743 (46.5) | 0.082 |
| MALES, No. (%) | 1085 (52.5) | 229 (48.8) | 856 (53.5) | 0.082 |
| AIS, no. (%) | 1472 (71.2) | 371 (79.1) | 1101 (68.9) | < 0.001 |
| TIA, no. (%) | 596 (28.8) | 98 (20.9) | 498 (31.1) | < 0.001 |
| NIHSS, Mean (SD) | 3.5 (5.1) | 5.5 (6.4) | 2.9 (4.5) | < 0.001 |
| NIHSS, Median (IQR) | 2.0 (0.0 - 4.0) | 3.0 (1.0 - 8.0) | 1.0 (0.0 - 4.0) | < 0.001 |
| mRS admission, Mean (SD) | 1.9 (1.7) | 2.6 (1.7) | 1.7 (1.6) | < 0.001 |
| mRS admission, Median (IQR) | 2.0 (0.0 - 3.0) | 3.0 (1.0 - 4.0) | 1.0 (0.0 - 3.0) | < 0.001 |
| mRS release, Mean (SD) | 1.4 (1.6) | 2.1 (1.8) | 1.2 (1.5) | < 0.001 |
| mRS release, Median (IQR) | 1.0 (0.0 - 2.0) | 2.0 (0.0 - 4.0) | 1.0 (0.0 - 2.0) | < 0.001 |
| Ipsilateral ICA-Stenosis > 50%, no. (%) | 130 (6.3) | 30 (6.4) | 100 (6.3) | 0.997 |
| Hypertension, no. (%) | 1571 (76.0) | 385 (82.1) | 1186 (74.2) | < 0.001 |
| Diabetes, no. (%) | 469 (22.7) | 122 (26.0) | 347 (21.7) | 0.058 |
| Heart failure, no. (%) | 241 (11.7) | 108 (23.0) | 133 (8.3) | < 0.001 |
| Coronary artery disease (CAD), no. (%) | 430 (20.8) | 132 (28.1) | 298 (18.6) | < 0.001 |
| Time on monitor [h], Mean (SD) | 47.2 (16.8) | 51.9 (16.2) | 45.9 (16.7) | < 0.001 |
| Effective monitoring time [h], Mean (SD) | 38.3 (14.7) | 42.4 (14.8) | 37.1 (14.4) | < 0.001 |
| AS5F, Mean (SD) | 67.0 (11.6) | 74.1 (9.4) | 64.9 (11.4) | < 0.001 |

##

## Table S3 Sex-Disaggregated Baseline Characteristics of the Derivation Dataset

|  | Total | Female | Male | p-value |
| --- | --- | --- | --- | --- |
| Patients | 1702 | 797 | 905 |  |
| AF, no. (%) | 103 (6.1) | 54 (6.8) | 49 (5.4) | 0,283 |
| Age, Mean (SD) | 71.4 (13.3) | 74.5 (13.0) | 68.7 (13.0) | < 0.001 |
| Age, Median (IQR) | 74.0 (63.0 - 81.0) | 78.0 (68.0 - 83.0) | 70.0 (60.0 - 79.0) | < 0.001 |
| AIS, no. (%) | 1196 (70.3) | 519 (65.1) | 677 (74.8) | < 0.001 |
| TIA, no. (%) | 506 (29.7) | 278 (34.9) | 228 (25.2) | < 0.001 |
| NIHSS, Mean (SD) | 3.2 (4.8) | 3.2 (5.0) | 3.1 (4.6) | 0,227 |
| NIHSS, Median (IQR) | 1.0 (0.0 - 4.0) | 1.0 (0.0 - 4.0) | 1.0 (0.0 - 4.0) | 0,227 |
| mRS admission, Mean (SD) | 1.8 (1.6) | 1.8 (1.7) | 1.8 (1.5) | 0,959 |
| mRS admission, Median (IQR) | 1.0 (0.0 - 3.0) | 1.0 (0.0 - 3.0) | 1.0 (0.0 - 3.0) | 0,959 |
| mRS release, Mean (SD) | 1.3 (1.5) | 1.4 (1.6) | 1.1 (1.4) | < 0.05 |
| mRS release, Median (IQR) | 1.0 (0.0 - 2.0) | 1.0 (0.0 - 2.0) | 1.0 (0.0 - 2.0) | < 0.05 |
| Ipsilateral ICA-Stenosis > 50%, no. (%) | 105 (6.2) | 28 (3.5) | 77 (8.5) | < 0.001 |
| Hypertension, no. (%) | 1265 (74.3) | 596 (74.8) | 669 (73.9) | 0,727 |
| Diabetes, no. (%) | 373 (21.9) | 157 (19.7) | 216 (23.9) | < 0.05 |
| Heart failure, no. (%) | 149 (8.8) | 69 (8.7) | 80 (8.8) | 0,963 |
| Coronary artery disease (CAD), no. (%) | 314 (18.4) | 104 (13.0) | 210 (23.2) | < 0.001 |
| Time on monitor [h], Mean (SD) | 46.5 (16.8) | 45.9 (16.6) | 47.0 (17.0) | 0,188 |
| Effective monitoring time [h], Mean (SD) | 37.5 (14.5) | 37.1 (14.5) | 37.9 (14.5) | 0,264 |
| AS5F, Mean (SD) | 65.4 (11.4) | 67.8 (11.6) | 63.3 (10.9) | < 0.001 |

## Table S4 Baseline Characteristics of the Sensitivity Analysis Dataset

###

|  | Total | AF | no AF |
| --- | --- | --- | --- |
| Patients | 1738 | 139 | 1599 |
| Pre-known AF, no. |  | 121 |  |
| AF diagnosed during index stay, no. |  | 18 |  |
| Age, Mean (SD) | 71.5 (13.2) | 78.5 (9.2) | 70.9 (13.4) |
| Age, Median (IQR) | 74.0 (63.0 - 81.0) | 81.0 (74.0 - 84.0) | 73.0 (62.0 - 81.0) |
| Females, no. (%) | 805 (46.3) | 62 (44.6) | 743 (46.5) |
| MALES, No. (%) | 933 (53.7) | 77 (55.4) | 856 (53.5) |
| AIS, no. (%) | 1198 (68.9) | 97 (69.8) | 1101 (68.9) |
| TIA, no. (%) | 540 (31.1) | 42 (30.2) | 498 (31.1) |
| NIHSS, Mean (SD) | 3.0 (4.6) | 3.7 (5.1) | 2.9 (4.5) |
| NIHSS, Median (IQR) | 1.0 (0.0 - 4.0) | 1.0 (0.0 - 4.5) | 1.0 (0.0 - 4.0) |
| mRS admission, Mean (SD) | 1.7 (1.6) | 2.0 (1.6) | 1.7 (1.6) |
| mRS admission, Median (IQR) | 1.0 (0.0 - 3.0) | 2.0 (1.0 - 3.0) | 1.0 (0.0 - 3.0) |
| mRS release, Mean (SD) | 1.2 (1.5) | 1.7 (1.8) | 1.2 (1.5) |
| mRS release, Median (IQR) | 1.0 (0.0 - 2.0) | 1.0 (0.0 - 3.0) | 1.0 (0.0 - 2.0) |
| Ipsilateral ICA-Stenosis > 50%, no. (%) | 150 (8.6) | 17 (12.2) | 133 (8.3) |
| Hypertension, no. (%) | 1300 (74.8) | 114 (82.0) | 1186 (74.2) |
| Diabetes, no. (%) | 385 (22.2) | 38 (27.3) | 347 (21.7) |
| Heart failure, no. (%) | 163 (9.4) | 30 (21.6) | 133 (8.3) |
| Coronary artery disease (CAD), no. (%) | 350 (20.1) | 52 (37.4) | 298 (18.6) |
| Troponin, Mean (SD) | 27.5 (93.1) | 57.4 (169.7) | 24.9 (82.8) |
| Glomerular filtration rate (GFR) [ml/min/1.73m²], Mean (SD) | 70.4 (21.0) | 58.5 (20.6) | 71.4 (20.7) |
| Time on monitor [h], Mean (SD) | 45.9 (16.8) | 46.4 (17.0) | 45.9 (16.7) |
| Effective monitoring time [h], Mean (SD) | 37.1 (14.5) | 37.8 (14.7) | 37.1 (14.4) |
| AS5F, Mean (SD) | 65.4 (11.3) | 71.4 (9.4) | 64.9 (11.4) |

##

## Table S5 Baseline Characteristics of the Internal Validation Dataset

|  | Total | AFib | no AFib |
| --- | --- | --- | --- |
| Patients | 397 | 87 | 310 |
| Pre-known AF, no. |  | 71 |  |
| AF diagnosed during index stay, no. |  | 16 |  |
| Age, Mean (SD) | 74.0 (15.2) | 81.2 (9.2) | 71.9 (15.9) |
| Age, Median (IQR) | 75.0 (65.0 - 84.0) | 83.0 (76.0 - 87.0) | 74.0 (63.0 - 82.0) |
| Females, no. (%) | 183 (46.1) | 44 (50.6) | 139 (44.8) |
| MALES, No. (%) | 214 (53.9) | 43 (49.4) | 171 (55.2) |
| AIS, no. (%) | 270 (68.0) | 60 (69.0) | 210 (67.7) |
| TIA, no. (%) | 127 (32.0) | 27 (31.0) | 100 (32.3) |
| NIHSS, Mean (SD) | 3.5 (5.0) | 4.6 (5.8) | 3.2 (4.6) |
| NIHSS, Median (IQR) | 2.0 (0.0 - 4.0) | 2.0 (0.0 - 5.5) | 1.0 (0.0 - 4.0) |
| mRS admission, Mean (SD) | 1.9 (1.6) | 2.5 (1.7) | 1.7 (1.5) |
| mRS admission, Median (IQR) | 1.0 (1.0 - 3.0) | 3.0 (1.0 - 4.0) | 1.0 (0.0 - 3.0) |
| mRS release, Mean (SD) | 1.4 (1.7) | 2.1 (1.9) | 1.3 (1.5) |
| mRS release, Median (IQR) | 1.0 (0.0 - 2.0) | 2.0 (0.0 - 3.5) | 1.0 (0.0 - 2.0) |
| Ipsilateral ICA-Stenosis > 50%, no. (%) | 38 (9.6) | 10 (11.5) | 28 (9.0) |
| Hypertension, no. (%) | 292 (73.6) | 72 (82.8) | 220 (71.0) |
| Diabetes, no. (%) | 84 (21.2) | 14 (16.1) | 70 (22.6) |
| Heart failure, no. (%) | 45 (11.3) | 20 (23.0) | 25 (8.1) |
| Coronary artery disease (CAD), no. (%) | 63 (15.9) | 24 (27.6) | 39 (12.6) |
| AS5F, Mean (SD) | 67.6 (13.0) | 73.7 (9.5) | 65.9 (13.3) |

##

## Table S6 Baseline Characteristics of the External Validation Dataset

|  | TOTAL | AF | NO AF | P-VALUE |
| --- | --- | --- | --- | --- |
| Patients | 1519 | 36 | 1483 |  |
| Age, Mean (SD) | 65.6 (12.9) | 74.8 (8.7) | 65.4 (12.9) | < 0.001 |
| Age, Median (IQR) | 66.0 (56.0 - 76.0) | 76.0 (69.0 - 79.0) | 66.0 (56.0 - 76.0) | < 0.001 |
| Females, no. (%) | 604 (39.8) | 16 (44.4) | 588 (39.6) | 0.683 |
| MALES, No. (%) | 915 (60.2) | 20 (55.6) | 895 (60.4) | 0.683 |
| NIHSS, Mean (SD) | 3.0 (3.0) | 3.4 (3.3) | 3.0 (3.0) | 0.470 |
| NIHSS, Median (IQR) | 2.0 (1.0 - 4.0) | 2.5 (1.0 - 4.0) | 2.0 (1.0 - 4.0) | 0.470 |
| AS5F, Mean (SD) | 57.6 (12.3) | 64.6 (7.3) | 57.4 (12.3) | < 0.001 |

##

## Table S7 Model Performance on the Derivation Dataset (Newly Detected AF Only) at a Specificity of 0.9

|  | AUC | Sensitivity | PPV | NPV | Accuracy | Threshold |
| --- | --- | --- | --- | --- | --- | --- |
| Neural Network | 0.86 [0.77, 0.94] | 0.65 [0.51, 0.73] | 0.46 [0.10, 0.72] | 0.97 [0.96, 1.00] | 0.79 [0.26, 0.96] | 0.18 |
| HRV | 0.87 [0.78, 0.95] | 0.66 [0.58, 0.75] | 0.27 [0.07, 0.53] | 0.78 [0.10, 0.98] | 0.70 [0.13, 0.94] | 0.11 |
| HRV + Clinical | 0.88 [0.79, 0.95] | 0.66 [0.52, 0.73] | 0.32 [0.13, 0.61] | 0.98 [0.96, 0.99] | 0.88 [0.82, 0.93] | 0.12 |
| HRV + Age | 0.88 [0.79, 0.95] | 0.66 [0.58, 0.72] | 0.31 [0.25, 0.38] | 0.98 [0.97, 0.98] | 0.89 [0.86, 0.91] | 0.12 |
| Clinical | 0.77 [0.68, 0.85] | 0.32 [0.27, 0.36] | 0.16 [0.12, 0.20] | 0.95 [0.95, 0.96] | 0.86 [0.84, 0.88] | 0.14 |
| Bayesian | 0.89 [0.80, 0.96] | 0.74 [0.58, 0.89] | 0.52 [0.11, 0.75] | 0.98 [0.97, 1.00] | 0.79 [0.23, 0.96] | 0.02 |
| AS5F | 0.72 [0.61, 0.81] | 0.28 [0.19, 0.38] | 0.15 [0.11, 0.20] | 0.95 [0.94, 0.96] | 0.86 [0.86, 0.88] | 78.82 |

##

## Table S8 Model Performance on the Derivation Dataset (Pre-Known and Newly Detected AF) at a Specificity of 0.9

|  | AUC | Sensitivity | PPV | NPV | Accuracy | Threshold |
| --- | --- | --- | --- | --- | --- | --- |
| Neural Network | 0.88 [0.84, 0.92] | 0.69 [0.64, 0.75] | 0.68 [0.52, 0.82] | 0.91 [0.88, 0.94] | 0.84 [0.79, 0.88] | 0.42 |
| HRV | 0.89 [0.86, 0.93] | 0.71 [0.60, 0.80] | 0.69 [0.62, 0.74] | 0.92 [0.90, 0.94] | 0.86 [0.83, 0.89] | 0.24 |
| HRV + Clinical | 0.90 [0.86, 0.93] | 0.73 [0.63, 0.81] | 0.69 [0.64, 0.72] | 0.92 [0.89, 0.94] | 0.86 [0.84, 0.88] | 0.26 |
| HRV + Age | 0.90 [0.86, 0.93] | 0.71 [0.60, 0.81] | 0.69 [0.63, 0.71] | 0.92 [0.91, 0.94] | 0.86 [0.84, 0.88] | 0.26 |
| Clinical | 0.77 [0.71, 0.81] | 0.35 [0.30, 0.38] | 0.51 [0.48, 0.52] | 0.83 [0.82, 0.85] | 0.78 [0.77, 0.78] | 0.40 |
| Bayesian | 0.92 [0.88, 0.95] | 0.77 [0.71, 0.81] | 0.70 [0.61, 0.83] | 0.93 [0.91, 0.94] | 0.87 [0.84, 0.91] | 0.08 |
| AS5F | 0.73 [0.68, 0.79] | 0.31 [0.26, 0.36] | 0.46 [0.42, 0.49] | 0.81 [0.80, 0.82] | 0.76 [0.76, 0.77] | 78.86 |

##

## Table S9 Model Performance on the Derivation Dataset (Newly Detected AF Only) at Youden’s J

|  | AUC | Sensitivity | Specificity | PPV | NPV | Accuracy | Threshold |
| --- | --- | --- | --- | --- | --- | --- | --- |
| Neural Network | 0.86 [0.77, 0.94] | 0.80 [0.71, 0.91] | 0.84 [0.81, 0.89] | 0.40 [0.09, 0.63] | 0.97 [0.96, 1.00] | 0.77 [0.21, 0.95] | 0.15 |
| HRV | 0.87 [0.78, 0.95] | 0.81 [0.72, 0.91] | 0.86 [0.75, 0.92] | 0.35 [0.22, 0.56] | 0.97 [0.97, 0.98] | 0.90 [0.85, 0.95] | 0.12 |
| HRV + Clinical | 0.88 [0.79, 0.95] | 0.84 [0.79, 0.89] | 0.84 [0.74, 0.88] | 0.31 [0.13, 0.57] | 0.98 [0.96, 0.99] | 0.87 [0.82, 0.93] | 0.12 |
| HRV + Age | 0.88 [0.79, 0.95] | 0.83 [0.77, 0.91] | 0.84 [0.77, 0.89] | 0.33 [0.26, 0.41] | 0.98 [0.97, 0.98] | 0.90 [0.86, 0.92] | 0.13 |
| Clinical | 0.77 [0.68, 0.85] | 0.89 [0.82, 0.95] | 0.63 [0.58, 0.68] | 0.12 [0.09, 0.14] | 0.98 [0.96, 0.99] | 0.62 [0.51, 0.71] | 0.11 |
| Bayesian | 0.89 [0.80, 0.96] | 0.84 [0.78, 0.93] | 0.88 [0.82, 0.91] | 0.50 [0.11, 0.76] | 0.98 [0.97, 1.00] | 0.79 [0.21, 0.96] | 0.02 |
| AS5F | 0.72 [0.61, 0.81] | 0.81 [0.67, 0.98] | 0.56 [0.32, 0.72] | 0.10 [0.08, 0.10] | 0.97 [0.96, 0.98] | 0.56 [0.55, 0.58] | 67.73 |

##

## Table S10 Model Performance on the Derivation Dataset (Pre-Known and Newly Detected AF) at Youden’s J

|  | AUC | Sensitivity | Specificity | PPV | NPV | Accuracy | Threshold |
| --- | --- | --- | --- | --- | --- | --- | --- |
| Neural Network | 0.88 [0.84, 0.92] | 0.81 [0.78, 0.85] | 0.85 [0.77, 0.89] | 0.61 [0.45, 0.74] | 0.92 [0.90, 0.94] | 0.81 [0.72, 0.87] | 0.35 |
| HRV | 0.89 [0.86, 0.93] | 0.77 [0.72, 0.82] | 0.89 [0.82, 0.94] | 0.71 [0.62, 0.76] | 0.91 [0.89, 0.94] | 0.87 [0.83, 0.90] | 0.26 |
| HRV + Clinical | 0.90 [0.86, 0.93] | 0.80 [0.75, 0.84] | 0.87 [0.77, 0.92] | 0.66 [0.61, 0.71] | 0.92 [0.90, 0.95] | 0.86 [0.83, 0.88] | 0.23 |
| HRV + Age | 0.90 [0.86, 0.93] | 0.79 [0.73, 0.84] | 0.87 [0.78, 0.94] | 0.68 [0.62, 0.71] | 0.92 [0.91, 0.94] | 0.86 [0.83, 0.88] | 0.24 |
| Clinical | 0.77 [0.71, 0.81] | 0.78 [0.74, 0.84] | 0.66 [0.61, 0.74] | 0.38 [0.36, 0.42] | 0.89 [0.86, 0.93] | 0.67 [0.63, 0.71] | 0.26 |
| Bayesian | 0.92 [0.88, 0.95] | 0.82 [0.76, 0.87] | 0.89 [0.84, 0.94] | 0.71 [0.63, 0.83] | 0.93 [0.90, 0.94] | 0.87 [0.85, 0.91] | 0.08 |
| AS5F | 0.73 [0.68, 0.79] | 0.79 [0.63, 0.92] | 0.57 [0.42, 0.69] | 0.34 [0.32, 0.36] | 0.89 [0.86, 0.92] | 0.60 [0.59, 0.63] | 68.06 |

##

## Table S11 Model Performance on the External Validation Dataset at Pre-defined Threshold

|  | AUC | Sensitivity | Specificity | PPV | NPV | Threshold |
| --- | --- | --- | --- | --- | --- | --- |
| Mondafis (1h) | 0.79 [0.73, 0.85] | 0.39 [0.23, 0.56] | 0.89 [0.88, 0.91] | 0.08 [0.04, 0.13] | 0.98 [0.98, 0.99] | 0.29 |
| AS5F | 0.68 [0.61, 0.75] | 0.03 [0.00, 0.09] | 0.96 [0.95, 0.97] | 0.02 [0.00, 0.06] | 0.98 [0.97, 0.98] | 78.79 |

##

## Table S12 Model Performance on the External Validation Dataset at Youden’s J

|  | AUC | Sensitivity | Specificity | PPV | NPV | Threshold |
| --- | --- | --- | --- | --- | --- | --- |
| Mondafis (1h) | 0.79 [0.73, 0.85] | 0.88 [0.69, 1.00] | 0.62 [0.45, 0.80] | 0.06 [0.03, 0.09] | 1.0 [0.99, 1.00] | 0.10 |
| AS5F | 0.68 [0.61, 0.75] | 0.86 [0.59, 1.00] | 0.51 [0.32, 0.76] | 0.04 [0.03, 0.06] | 0.99 [0.99, 1.00] | 58.61 |

## Table S13 2x2 Table of the External Validation Run

| Total: 1519 Patients | AF Predicted (164) | No AF Predicted (1355) |
| --- | --- | --- |
| AF Diagnosis (36) | **13 (True Positives)** | **23 (False Negatives)** |
| No AF Diagnosis (1483) | **151 (False Positives)** | **1332 (True Negatives)** |
