# Supplementary material for: Artificial intelligence for prediction of atrial fibrillation in the stroke unit: a retrospective derivation validation cohort study
Source: eBioMedicine. 2025 Aug 5;118:105869. doi: 10.1016/j.ebiom.2025.105869 (PMC12341230; doi:10.1016/j.ebiom.2025.105869)
Supplement: Caption for Supplementary Figures [file mmc9.docx]

## **Figure Captions for Supplementary Materials**

**Fig. S1 Deep Neural Network Architecture.** Convolutional neural network architecture adapted from Attia et al. (reference No. 12) that processes input data of one ECG channel through a series of convolutional blocks. The network consists of three "Multiple Residual Blocks" with 16, 32, and 64 filters respectively, using kernel sizes of 7, 5, and 3. Each of these blocks is followed by dropout layers with a rate of 0.2. The architecture then applies a 1D convolution with 128 filters (k=1), batch normalization, Rectified Linear Unit (ReLU) activation, and another dropout layer before flattening the features. The network concludes with a dense layer using sigmoid activation for the final output. The detailed block structure (shown on the right) reveals the internal components of a residual block, which includes convolutional layers, max pooling, batch normalization, and ReLU activations arranged in a sequential manner.

**Fig. S2 Comparative Testing of Established AF Risk Scores.** Testing was performed on the derivation dataset (1702 patients, thereof 106 with newly detected AF). 28 patients had to be excluded additionally due to missing triglyceride and cholesterol levels. All curves are plotted with 95% confidence intervals. ROC-AUCs are provided in the legend. ROC: Receiver operating characteristic; AUC: Area under the curve.

**Fig. S3 Results of Comparative Testing of Different Models on the Sensitivity Analysis Dataset.** a) Schematic depiction of the different classes of features and models. b, c, d) ROC curves of neural network, ensemble and fusion models. All curves are plotted with 95% confidence intervals across folds. ROC-AUCs and respective 95% confidence intervals are provided in the legends. ROC: Receiver operating characteristic; AUC: Area under the curve; HRV: Heart rate variability.

**Fig. S4 ROC-AUCs of the Simplified Model by Duration of CEM Data Used for HRV Calculation.** Dataset included patients without AF and newly detected AF. ROC: Receiver operating characteristic; AUC: Area under the curve; CEM: Continuous ECG monitoring. HRV: heart rate variability.

**Fig. S5 ROC-AUCs of Internal Validation Analysis of the Final Model.** Dataset included patients without AF and newly detected AF. ROC: Receiver operating characteristic; AUC: Area under the curve; AF: Atrial Fibrillation.

**Fig. S6 Comparative Testing of Established AF Risk Scores on the External Validation Dataset.** The analysis was performed on a subset of the external validation dataset consisting of 1,507 patients, including 36 patients with newly detected atrial fibrillation (AF). Twelve patients were excluded from the original dataset due to missing concomitant diagnoses. ROC curves are shown with 95% confidence intervals, and corresponding ROC-AUC values are provided in the legend. ROC: Receiver operating characteristic; AUC: Area under the curve.

**Fig. S7 ROC-AUCs of External Validation Analysis with Atrial Runs Removed from the CEM Data.** Hand-labelled short atrial runs, along with a buffer of 30 seconds before and after each run, were excluded from the continuous ECG monitoring dataset prior to analysis. One patient was excluded due to insufficient analysable data remaining after this removal. ROC: Receiver operating characteristic; AUC: Area under the curve; CEM: continuous ECG monitoring.
