# Supplementary material for: Artificial intelligence for prediction of atrial fibrillation in the stroke unit: a retrospective derivation validation cohort study
Source: eBioMedicine. 2025 Aug 5;118:105869. doi: 10.1016/j.ebiom.2025.105869 (PMC12341230; doi:10.1016/j.ebiom.2025.105869)
Supplement: Supplementary Results [file mmc10.docx]

# Supplementary Results

## Additional Analysis Including Patients with Pre-Known AF

Out of the remaining 2,068 patients (47.5 % females, mean age 73.1 years), 469 (22.7 %) were diagnosed with atrial fibrillation (AF; 366 with pre-known AF diagnosis, 106 with diagnosis during index hospital stay, Supplementary Table S2).

The AS5F score achieved a ROC-AUC of 0.73 [95% Confidence Interval (CI) 0.68-0.79]. We first compared it to an ensemble machine learning (ML) model using multimodal clinical data, including baseline characteristics, concomitant diagnoses, and laboratory values. This model achieved a ROC-AUC of 0.77 ([0.71, 0.81], p=1.96e-04; Figure 3 F).

The morphologic properties of ECG in sinus rhythm contain information about underlying atrial disease and may thus inform prediction models. Therefore, we next trained and tested a deep neural network (DNN) on 5.12-second continuous electrocardiogram monitoring (CEM) segments. We observed a general trend where the inclusion of more segments increased performance up to 20 segments (Figure 3 B inset). A DNN utilizing 20 segments significantly outperformed the AS5F score (ROC-AUC 0.88 [0.84, 0.92], p=1.83e-26; Figure 3 E).

Long-term patterns in CEM signals captured by heart rate variability (HRV) metrics may contain complementary information to that represented by p-wave and QRS complex morphology. HRV-based models have consequently been used to identify patients at high risk for AF. Here, an ensemble ML model using HRV metrics as input achieved a ROC-AUC of 0.89 [0.86, 0.93], significantly outperforming both the clinical ML model and the AS5F score (p=4.73e-22 and p=4.58e-32, respectively) but not the DNN (p=6.13e-02). We next evaluated whether including multimodal clinical variables in addition to HRV would further improve predictions. This approach achieved a slightly higher ROC-AUC of 0.90 [0.86, 0.93] than HRV alone or the DNN (p=3.47e-02 and p=2.66e-02, respectively).

Considering that DNNs assess morphological features from raw CEM data which may contain complementary information to long-term trends in HRV, we next assessed performance of a Bayesian fusion model combining both DNN on raw CEM data and ensemble ML models analysing HRV, along with clinical information. This model achieved a ROC-AUC of 0.92 [0.88, 0.95] (Figure 3 G), outperforming both, the DNN (p=2.17e-09) and the ensemble ML models using HRV or HRV and clinical data (p=2.42e-04 and p=1.13e-02, respectively).

## Additional Sensitivity Analyses

To assess the robustness of our predictions, we performed a sensitivity analysis using a subset of our data that included controls and patients diagnosed with AF (newly detected or pre-known) but without AF alerts during monitoring. The AF patients in this subset were generally younger, had fewer comorbidities, and were less severely affected than those in the original dataset (Supplement Table S4). While ROC-AUCs were generally lower (ROC-AUC of the best-performing Bayesian fusion model 0.78 [0.68-0.86]), findings regarding the hierarchy of performance of different approaches generally remained the same. ECG-based models consistently outperformed the AS5F (Supplementary Figure S3).

## Explainability: Insights from the Sensitivity Analysis

When trained and tested on a subset of our data that contained healthy controls and patients with AF diagnosis but no AF-alerts during monitoring (and therefore presumably also less arrhythmic events than the original dataset), the DNN's predictions were comparable to the AS5F-Score (ROC-AUC 0.71 [0.60, 0.81] vs. 0.67 [0.58, 0.76], p=3.36e-01). Meanwhile, a HRV and clinical feature-based ensemble model still performed better than chance and better than the AS5F-Score on this subset of data (ROC-AUC 0.77 [0.67, 0.85] vs. 0.67 [0.58, 0.76], p=1.24e-03, Supplementary Figure S3). Baseline characteristics of the sensitivity analysis cohort are provided in Supplementary Table S4.
